# Lateral π-extended helical nanographenes with large spin polarization

**DOI:** 10.1039/d5sc03887a

**Published:** 2025-08-13

**Authors:** Wenhui Niu, Chi Fang, Likun Tang, Elif Unsal, Yubin Fu, Jitul Deka, Fupin Liu, Alexey A. Popov, Fupeng Wu, Huanhuan Shi, Hartmut Komber, Arezoo Dianat, Rafael Gutierrez, Ji Ma, Yutao Sang, Gianaurelio Cuniberti, Stuart S. P. Parkin

**Affiliations:** a Max Planck Institute of Microstructure Physics Weinberg 2 06120 Halle Germany wenhui.niu@mpi-halle.mpg.de stuart.parkin@mpi-halle.mpg.de; b Center for Advancing Electronics Dresden (cfaed) & Faculty of Chemistry and Food Chemistry, Technische Universität Dresden Mommsenstraße 4 01062 Dresden Germany; c State Key Laboratory of Molecular Engineering of Polymers, Department of Macromolecular Science, Fudan University 200438 Shanghai P. R. China sangyt@fudan.edu.cn; d Institute for Materials Science and Max Bergmann Center of Biomaterials, TU Dresden 01062 Dresden Germany gianaurelio.cuniberti@tu-dresden.de; e Leibniz Institute for Solid State and Materials Research (IFW Dresden) Helmholtzstraße 20 01069 Dresden Germany; f Jiangsu Key Laboratory of New Power Batteries, Jiangsu Collaborative Innovation Center of Biomedical Functional Materials, School of Chemistry and Materials Science, Nanjing Normal University 210023 Nanjing P. R. China; g Max Planck Institute Chemical Physics of Solids Nöthnitzer Straße 40 01187 Dresden Germany; h Karlsruhe Institute of Technology, Institute for Quantum Materials and Technologies Kaiserstraße 12 76131 Karlsruhe Germany; i Leibniz-Institut für Polymerforschung Dresden e. V. Hohe Straße 6 01069 Dresden Germany; j College of Materials Science and Opto-Electronic Technology & Center of Materials Science and Optoelectronics Engineering, University of Chinese Academy of Science 100049 Beijing P. R. China; k Dresden Center for Computational Materials Science (DCMS), TU Dresden 01062 Dresden Germany

## Abstract

The possibility that current passing through an organic molecule becomes spin-polarized is highly intriguing. Amongst these molecules, helicene units have recently been shown to exhibit such a chiral-induced spin selectivity (CISS) effect. Thus, helical nanographenes (NGs), whose core building block is a helicene unit, are natural candidates for generating CISS. However, reports on the CISS effect in helical nanographenes (NGs) remain limited, primarily due to the lack of a suitable molecular platform for detecting spin-selective transport. In this work, we have developed a synthetic strategy using pre-fused key bonds in oligophenylene precursors and successfully synthesized lateral extended NGs that incorporate either single or double undecabenzo[7]helicene units with high yields. The resultant lateral extended helical NGs display excellent chiroptical properties including strong circular dichroism and large dissymmetry factors. Furthermore, magneto-conductive atomic force microscopy (mc-AFM) and magnetoresistance (MR) measurements show clear evidence for spin polarization of the current with a large spin polarization of up to 80% and a robust MR of 1.5% at room temperature. Together with theoretical modeling, our results identify lateral extended helical NGs as promising quantum materials for future organic spintronic devices.

## Abstract 2

Lateral extended helical nanographenes (NGs) embedded with undecabenzo[7]helicene exhibit significant spin polarization (up to 80%) as well as robust magnetoresistance (1.5%), highlighting their potential for molecular spintronics applications.

## Graphical abstract

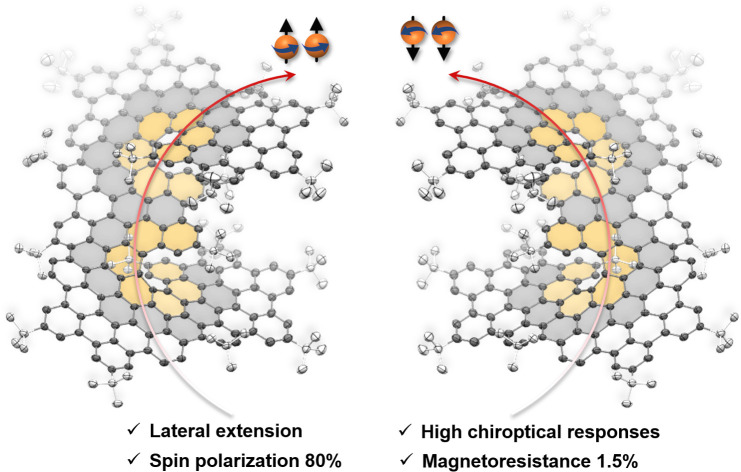

## Introduction

The chiral-induced spin selectivity (CISS) effect is a phenomenon whereby the transmission of electron spin through chiral matter, in the absence of any external magnetic fields, depends on their chirality. This leads to an imbalance in the number of up and down spin electrons and the current is, thus, spin-polarized.^1–3^ The CISS effect was first observed as the spin-dependent transmission of photoelectrons through monolayers of chiral fatty acid stearoyl-lysine.^4^ Subsequently, the effect was explored in a wide array of molecular,^5,6^ supramolecular,^7,8^ and other materials.^9–11^ Helicenes, a classic family of chiral molecules, therefore, are natural candidates for expressing the CISS effect.^12^ Indeed, thia[4]helicene on a metal surface displays a large spin polarization of ∼60% at low voltages,^13^ and carbo[6]helicenes have been recently reported to display spin polarizations ranging from 35% to 80%, depending on their different substitutions.^14,15^

The high spin polarization of current transmitted through chiral molecules makes them of great interest for future applications as spin injectors for quantum applications^16^ and for next-generation information storage technologies.^17,18^ The further development of molecular spintronics requires both high spin polarization and large spin polarized currents and, accordingly, there have been significant efforts to achieve these goals over the past few years. An important result from studies of DNA and oligopeptides was that the spin filtering of the charge current increases in proportion to the length of the molecules.^19,20^ Another important finding was a qualitative correlation between the intensity of the lowest energy Cotton peak in circular dichroism (CD) and the spin polarization resulting from the CISS effect, indicating that intense chiroptical responses might be responsible for high spin polarizations.^21^

As compared to helicenes, helical nanographenes (NGs), composed of extended nanographene motifs embedded with [*n*]helicene units, have a larger conjugated backbone, allowing for increased chiroptical responses.^22–25^ In addition, lateral π-extension, that gives rise to extended electron delocalization, could reduce the energy gaps and enhance the absorption or CD intensities,^26–31^ making this strategy promising for yielding suitable molecules optimized for high spin polarization and large spin polarized currents.^32^

**Scheme 1:**
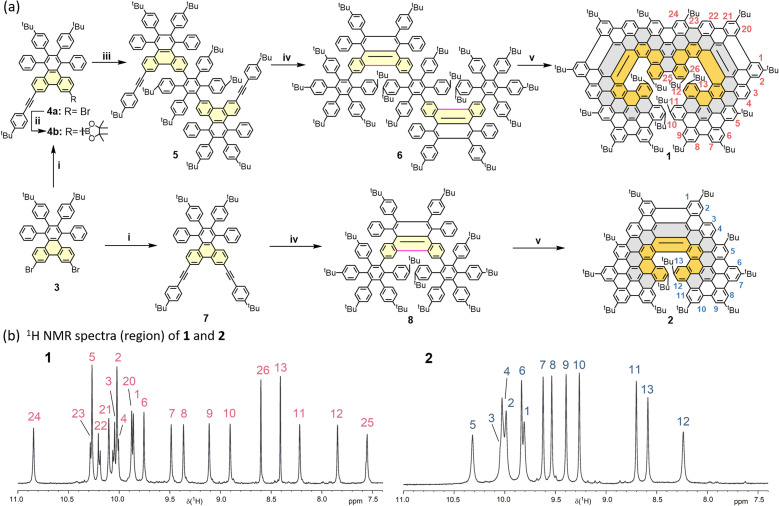
(a) Synthetic route to lateral extended double and single undecabenzo[7]helicenes (1 and 2). (i) 4-(*Tert*-butyl)phenylacetylene, Pd(PPh_3_)_2_Cl_2_, CuI, THF, Et_3_N, 80 °C, 24 h, 20% for 4a; 10% for 7; (ii) Pd(dppf)Cl_2_, KOAc, bis(pinacolato)diboron, dioxane, 85 °C, 18 h, 61% for 4b; (iii) Pd(PPh_3_)_4_, K_2_CO_3_, dioxane, H_2_O, 95 °C, 24 h, 49%; (iv) tetrakis(4-(*tert*-butyl)phenyl)cyclopenta-2,4-dien-1-one, Ph_2_O, 270 °C, 24 h, 80% for 6, and 84% for 8; (v) DDQ, DCM, TfOH, 0 °C, 40 min, 41% for 1, and 73% for 2. (b) ^1^H NMR spectra (region of aromatic protons) of 1 and 2 (solvent: C_2_D_2_Cl_4_, 30 °C).

Here, we report the successful synthesis of lateral π-extended helical NGs 1 and 2 containing double and single undecabenzo[7]helicenes, respectively. Previously, the lateral extended helical NGs 1 and 2 have been achieved with yields of 18%^27^ and 4%,^30^ respectively, mainly owing to the flexible precursors design, leading to the high strain to prevent the further fusion of the inner bonds once the outer hexabenzocoronene units formed during the Scholl reaction. In this work, we have developed new synthetic strategy to pre-fuse these key bonds (highlighted in pink, Scheme 1a) in oligophenylene precursors to avoid the strain, therefore, resulting in the regioselective Scholl reaction with high yields of 41% for 1 and 73% for 2. The embedded undecabenzo[7]helicene units provide inherent chirality, allowing their chiral resolution and the investigation of chiroptical properties. Notably, the enantiomers of lateral extended helical NG 1-*PP*/*MM* present strong chiroptical activities with a Cotton effect of 357 M^−1^ cm^−1^ at 556 nm and a large dissymmetry factor (|*g*_abs_|) of 10.6 × 10^−3^ at 598 nm. Employing mc-AFM and MR measurements, we disclose a clear CISS behavior with a spin polarization of ∼80% along with a spin-polarized current of ∼20 nA as well as a robust MR of 1.5% at room temperature, making this class of lateral extended helical NGs promising candidates as molecular spin filters.

## Results and discussion

The synthetic route to lateral extended helical NGs 1 and 2 is depicted in Scheme 1a. First, the 7,10-dibromo-2,3-bis(4-(*tert*-butyl)phenyl)-1,4-diphenyltriphenylene (4a) and 7,10-dibromo-2,3-bis(4-(*tert*-butyl)phenyl)-1,4-diphenyltriphenylene (7) were synthesized by one-fold and two-fold Sonogashira coupling between 7,10-dibromo-2,3-bis(4-(*tert*-butyl)phenyl)-1,4-diphenyltriphenylene (3) and 4-(*tert*-butyl)phenylacetylene. Compound 2-(6,7-bis(4-(*tert*-butyl)phenyl)-11-((4-(*tert*-butyl)phenyl)ethynyl)-5,8-diphenyltriphenylen-2-yl)-4,4,5,5-tetramethyl-1,3,2-dioxaborolane (4b) was prepared in 61% yield by borylation reaction from 4a. Then, the two-fold Suzuki coupling of 4,4′′-di-*tert*-butyl-4′,5′-bis(4-(*tert*-butyl)phenyl)-3′,6′-diiodo-1,1′:2′,1′′-terphenyl and compound 4b gave 10,10′-(4,4′′-di-*tert*-butyl-4′,5′-bis(4-(*tert*-butyl)phenyl)-[1,1′:2′,1′′-terphenyl]-3′,6′-diyl)bis(2,3-bis(4-(*tert*-butyl)phenyl)-7-((4-(*tert*-butyl)phenyl)ethynyl)-1,4-diphenyltriphenylene) (5) in 49% yield. Subsequently, Diels–Alder reaction between 2,3,4,5-tetrakis(4-(*tert*-butyl)-phenyl)cyclopenta-2,4-dien-1-one and compound 5 or 7 yielded the corresponding oligophenylene precursors 6 or 8 in high yields (80–84%). At last, Scholl reaction of precursors 6 or 8 treated by 2,3-dichloro-5,6-dicyano-1,4-benzo-quinone (DDQ) and trifluoromethanesulfonic acid (TfOH), gave the target lateral extended helical NGs embedded with double undecabenzo[7]helicene (1) in 41% yield or single undecabenzo[7]helicene (2) in 73% yield, respectively. It is noteworthy that during the intramolecular Scholl reaction, there are 29 and 19 carbon–carbon (C–C) bonds newly formed, making the average yields for each new C–C bond formation approximately 97% for 1 and 98.5% for 2, further confirming the high efficiency and regioselectivity of the Scholl reaction attributed to the pre-fused key bonds (highlighted in pink) in precursors 6 and 8.

The structures of the obtained undecabenzo[7]helicene derivatives were initially confirmed using high-resolution matrix-assisted laser desorption/ionization time-of-flight (MALDI-TOF) mass spectrometry (Fig. S22 and S28). Both helical NGs 1 and 2 display consistent mass spectra in comparison with the calculated isotopic distribution patterns. Later on, NMR analysis was performed to further confirm their chemical structures. The ^1^H NMR spectra align well with the anticipated structures of helical NGs 1 and 2, and all signals have been successfully assigned with the help of 2D NMR techniques (Fig. S23–S27 and S29–S33). Characteristically, the inner protons 11–13 (also 25 for 1) of both helical NGs show a significant shielding compared to the similar but outer protons 6–10, due to the screw-type layered structure (Scheme 1b), in good agreement with the NMR analyses of reported NGs.^27,30^

**Fig. 1:**
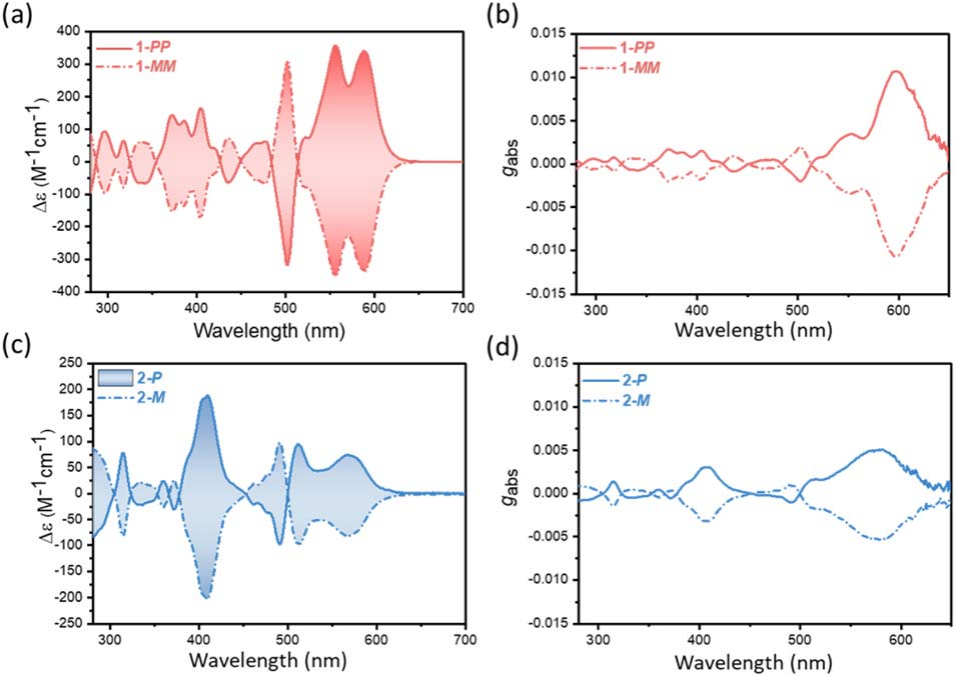
(a and c) Experimental CD spectra of the pure enantiomers of 1-*PP*/*MM* and 2-*P*/*M* in anhydrous DCM. (b and d) *g*_abs_ of pure enantiomers 1-*PP*/*MM* and 2-*P*/*M* recorded in anhydrous DCM.

The rigid backbone and stable chirality of the undecabenzo[7]helicenes 1 and 2 allow us to achieve their racemic resolution by chiral high performance liquid chromatography (HPLC). The successful chiral resolution yielded the optically pure 1-*PP* and 1-*MM* as well as 2-*P* and 2-*M*, respectively (Fig. S36–37), with the chirality of each enantiomer assigned compared with the reported cases.^27,30^ Subsequently, the chiroptical properties of the pure enantiomers were investigated by CD spectroscopy recorded in anhydrous DCM. The enantiomers of the lateral extended helical NGs 1 and 2 exhibit pronounced CD activity across the 300–650 nm range. Remarkably, the double undecabenzo[7]helicene 1 present intensive Cotton effects of 357 M^−1^ cm^−1^ at 556 nm and 340 M^−1^ cm^−1^ at 588 nm (Fig. 1a), stronger than the Cotton effects at 94 M^−1^ cm^−1^ at 510 nm and 76 M^−1^ cm^−1^ at 568 nm of single undecabenzo[7]helicene 2 (Fig. 1c). In addition, the double undecabenzo[7]helicene 1 displays upgraded |*g*_abs_| of 10.6 × 10^−3^ at 598 nm (Fig. 1b), two-fold higher than the |*g*_abs_| value of 5.0 × 10^−3^ at 582 nm of 2-*P*/*M* (Fig. 1d), in good alignment with reported values.^27,30^ Notably, compared to its non-extended counterpart,^24^ the lateral extension of the M-shaped double undecabenzo[7]helicene 1 has been found to remain the precise alignment of electric and magnetic transition dipole moments (Fig. S40), surpassing the chiroptical properties of its isomer with X-shaped double undecabenzo[7]helicene (5.0 × 10^−3^ at 430 nm).^27^

**Fig. 2:**
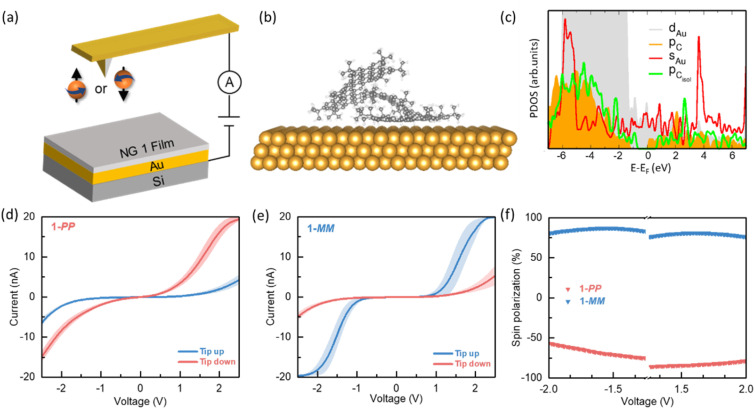
Spin polarization measurements of helical NG 1. (a) Schematic diagram of mc-AFM measurements. (b) Side view of the 1-*PP* molecule on a Au(111) surface after structural optimization. The *tert*-butyl groups closed to the substrate are pushed away from it due to the vdW interactions. (c) Projected, orbital-resolved electronic density of states (PDOS) in arbitrary units for the 6s and 5d gold orbitals as well as for the 2p carbon orbitals. *p*_Cisol_ denotes the 2p states for the molecule in the gas phase. The average current as a function of applied voltage for (d) 1-*PP* and (e) 1-*MM* when the tip is magnetized in the upward (blue line) and downward (red line) directions. (f) Spin polarization values of 1-*PP* and 1-*MM* as a function of applied bias voltage.

Previous work suggests that the high CD intensity of chiral molecules correlates with a high spin polarization which indicates that both phenomena relate to a similar underlying microscopic mechanism, namely, correlated electric and magnetic density fluctuations.^21,33^ In our system, the inherent chirality and rigid backbone together with the intensive chiroptical responses make lateral extended helical NG 1 as a promising molecular platform for spin-selective electron transport. To evaluate the spin polarization, we first conducted mc-AFM where the NG 1 layer lay on the gold surface and the pre-magnetized tip was used to inject the spin polarized electrons (Fig. 2a). Considering the structure of NGs absorbed on a gold substrate, we have computed basic structural and electronic features of the NG 1 on a Au(111) surface (only the *PP* enantiomer was addressed, since the *MM* enantiomer displays a similar behavior due to the symmetry). Because of the relatively complex molecular structure of 1, we simulated three initial configurations of the molecule 1 with respect to the metallic substrate (gold): initial average separation between the closest C atom and an Au atom underneath being (a) 3.7 Å, (b) 1.90 Å, and (c) 1.90 Å with the molecule rotated by 5° around the axis perpendicular to the surface. All these structures relaxed approximately to the same final configuration shown in Fig. 2b (corresponding to case (b) above), where the *tert*-butyl groups closest to the surface were pushed away from it due to van-der-Waals (vdW) interactions. As a result of the vdW interactions between the largely delocalized conjugated area and the gold atomic surface, the laterally extended helical NG 1 present a face-on conformation on the surface, beneficial to the potential spin polarization behavior. The distances between C atoms belonging to the basal plane and Au atoms ranged from 3.22 Å to 3.36 Å, lying within the typical vdW distances. Independent of the initial geometry and the used vdW corrections (D3 and TS), we found a charge transfer of 0.3*e* from the molecule to the substrate. The projected, orbital-resolved electronic density of states (PDOS) shows a molecular HOMO–LUMO gap of approximately 1.5 eV, which is underestimated due to the well-known problems of density-functional theory, inherited by the parametrized tight-binding approach used in these calculations (see Section 6 in the SI). The Fermi level of the system is very close to the bottom of the conduction states, which is compatible with the charge transfer to the substrate (Fig. 2c). For comparison, the PDOS arising from the valence p-states of the molecule in the gas phase is also shown, indicating almost no change in the HOMO–LUMO gap, which is expected due to the weak physisorption on the substrate. The main difference to the isolated molecule is that the spectra of the 2p states are shifted relative to each other along the energy axis. This can be related to the charge transfer effects once the molecule is deposited on the gold substrate. We have also computed the adsorption energy of the molecule 1 on Au(111) and find that the TS dispersion correction leads to a stronger adsorption (−7.75 eV) than the D3 correction (−7.30 eV). The adsorption energy was computed as *E*_ads_ = *E*_mol+Au_ − *E*_mol_ − *E*_Au_, where *E*_X=mol+Au,mol,Au_ are the total energies of the corresponding subsystems. We also remark that slightly larger deviations in the final geometries of the three starting configurations mentioned above, only occur for parts of the molecules farther away from the basal plane (see Fig. S46 in the SI). Clearly, fluctuations in the rather flexible *tert*-butyl groups can lead to a variety of conformations differing only slightly in the spatial orientation of these groups, but the HOMO–LUMO gap, the average separation from the gold substrate, and the size of charge transfer are not expected to be dramatically influenced, as confirmed in the three studied configurations.

Encouraged by the simulated results, we conducted mc-AFM measurements with magnetization labeled as tip up when aligned with the magnet's north pole and tip down when aligned with the south pole (Fig. 2d and e). During the measurements, the conductive magnetic tips remained grounded, while the substrate potential was adjusted as required. In preparation for the experiments, NG 1-*PP* or 1-*MM* were dissolved in DCM, spin-coated onto a gold substrate to form a uniform thin film, and then annealed at 80 °C for 1 min. All tests were conducted at room temperature. AFM image of gold substrate before and after coating has been compared, suggesting a uniform coating of chiral molecules (Fig. S44). Fig. 2d and e details the relationship between current (*I*) and applied potentials (*V*) ranging from −2.5 to +2.5 V, depending on whether the magnetization of the tip is parallel (tip up) or antiparallel (tip down). Each error bar represents values averaged from at least three separate curves, with each curve calculated as the average of at least 30 *I*–*V* measurements taken at different locations. Specifically, the statistic averaged *I*–*V* curves for the 1-*PP* film show an increase in current with rising bias voltage when the tip is magnetized in the down direction (Fig. 2d). In contrast, when the tip is magnetized in the up direction, the overall current is significantly weaker, indicating that 1-*PP* exhibits greater resistance with tip up magnetization. For 1-*MM*, the observed current dependence on the magnetic orientation of the tip is reversed, suggesting that the *PP* and *MM* conformations of NG 1 have opposite spin selectivity. The mc-AFM results reveal distinct spin-polarized charge transport in the helical NGs, which is attributed to the CISS effect. Notably, NG 1 demonstrated highly spin polarized currents even at relatively low voltages, reaching the instrument's measurement threshold of 20 nA. This value substantially exceeds those reported for classic helicenes (typically 1–2 nA) as well as other chiral organic molecules such as DNA and oligopeptides (2–4 nA),^13–15^ which provides a significant advantage for their exploitation as promising quantum materials in future organic spintronics devices. The current spin polarization (SP) was defined as SP = (*I*_up_ – *I*_down_)/(*I*_up_ + *I*_down_) × 100%, where *I*_up_ and *I*_down_ represent the current values under a specific bias voltage when magnetized in the up and down directions, respectively. Calculations show that NG 1 exhibits an SP value of 80 ± 4% (Fig. 2f), more efficient than those of DNA of 35–65% dependent to length.^19^ In addition, the spin polarization measurements of NG 2 present a SP value of about 73% with spin polarized currents of 15 nA (Fig. S42), making this family of lateral extended helical NGs as excellent spin filters.

**Fig. 3:**
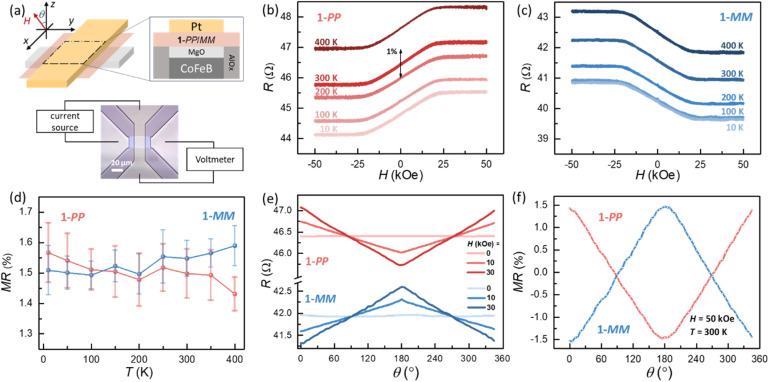
MR measurements in spin-valve-like devices based on 1-*PP*/*MM*. (a) Schematic device structure consisting of 1-*PP*/*MM* layer sandwiched by the bottom magnetic electrode and the top metal electrode orthogonally. One pair of bottom and top electrodes are connected to current source to load current *I* and the other pair is connected to the voltmeter to pick voltage *V*. Devices based on (b) 1-*PP* and (c) 1-*MM* showing a hysteresis loop in the magnetic field (*H*) dependence of the device resistance (*R* = *V*/*I*) at different temperatures. The black arrow indicates the MR magnitude of ±1%. (d) The temperature dependence of MR from (b) and (c). The error bar is given by the statistics of all data points (>200) with |*H*| > 45 kOe in each *R*–*H* curve. (e) Angle *θ* dependence of device resistance with an external field *H* = 0 to 30 kOe along + *z* direction at 300 K. (f) Angle *θ* dependence of MR with *H* = 50 kOe at 300 K.

A parallel test to evaluate the spin polarization behavior of lateral extended helical NGs was carried out by MR measurements where spin-valve like vertical devices were assembled (Fig. 3).^34^ Firstly, the bottom electrode was fabricated from a CoFeB (4 nm)\MgO (2.5 nm) extended film with in-plane spontaneous magnetization. Then, by spin-coating helical NG 1 and finally depositing a platinum capping layer, devices were made to characterize the electrical transport with a four-probe method (Fig. S45 for stepwise device fabrication). As shown in Fig. 3a, the bottom electrode and top electrode are patterned orthogonal to one another and isolated from each other by AlO_*x*_, which guarantees that the electrical currents in the top and bottom electrodes do not interfere with each other and only the junction resistance is reflected in the four-probe measurement. A charge current passing through the ferromagnetic CoFeB layer is spin-polarized along the magnetization orientation and then reaches the chiral NG layer after tunneling through the MgO layer. A CISS effect would indicate that the preferred spin direction in the chiral layer would yield a higher current (lower resistance), while the opposite spin direction is blocked, leading to a smaller current (higher resistance). Therefore, an external field tilts the magnetization towards the out-of-plane direction (±*z*-axis) and gives rise to a MR effect, that is quantified by MR = (*R*(*H*) − *R*(0))/*R*(0). Here, *R*(*H*) is the resistance in a magnetic field *H*, and *R*(0) is the resistance in zero magnetic field. As shown in Fig. 3b and c, the devices made from 1-*PP* and 1-*MM* display opposite field dependences of the MR, indicating opposite spin polarizations originating from the opposite chirality of the enantiomers. The MR values in both devices are about 1.5% at room temperature and remain nearly constant as the temperature varies from 10 to 400 K, indicating robust chirality-dependent spin-selective transport behavior in the NGs (Fig. 3d). To investigate the anisotropy of the CISS effect, we explored the angular dependence of the MR. A stable magnetic field (*H* = 0, 10, 30 kOe) is rotated from the *z*-axis by an angle *θ* (°) in the *yz*-plane, as defined in the Fig. 3a. The results shown in Fig. 3e indicate an out-of-plane unidirectional anisotropy of the CISS effect with a 360° period for devices made of 1-*PP* and 1-*MM*. As the external magnetic field increases, a larger proportion of the magnetization of the CoFeB layer is rotated.

Above the magnetic anisotropy field of CoFeB (∼20 kOe), the magnetization is saturated along the field and, thereby, maximizes the MR. As shown in Fig. 3f, the MR of the device made from 1-*PP* presents a maximum value of 1.5% at 0°, then gradually decreases to 0 at 90°, finally reaching −1.5% at 180°. For opposite chirality devices made by 1-*MM*, the sign of MR is reversed but otherwise exhibits a similar |MR| value and angular dependence.

**Fig. 4:**
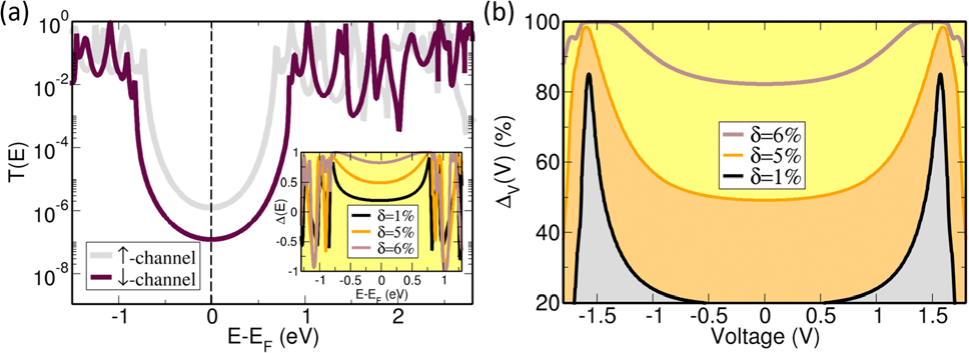
(a) Typical spin-dependent quantum mechanical transmission profile as a function of the electron injection energy for incoming spin-up and spin-down channels. The inset shows the spin asymmetry factor Δ(*E*) for various values of the parameter *δ* defined in the text. (b) Voltage-dependent integrated asymmetry factor Δ_V_(*V*) for different values of *δ*. Larger values of *δ* lead to a weak voltage dependence within the HOMO–LUMO energy window (approximately within the [−1, 1] voltage range).

To further understand the observed spin polarization behavior, we conducted theoretical calculations of the spin-dependent transmission probability of helical NG 1 using the Landauer formalism (Fig. 4). Details of the methodology and a more detailed description of the approximations used can be found in Section 6 of the SI. To quantify the spin polarization, we define a spin asymmetry factor Δ(*E*) = (*T*_↑_(*E*) − *T*_↓_(*E*))/(*T*_↑_(*E*) + *T*_↓_(*E*)). Here, ↑ and ↓ denote the two different computational (and experimental) setups, corresponding to the injection of spin polarized electrons from the magnetic tip, magnetized ↑ and ↓, into the molecule. A key quantity are the scaling factors *β*_↑_, *β*_↓_ for the spin-up and spin-down channels, see Section 6 of the SI, with which we define an asymmetry parameter, *δ* = |*β*_↑_ − *β*_↓_|/(*β*_↑_ + *β*_↓_) (see Section 6 in the SI). Values of *δ* = 1%, 5%, and 6% have been used for the transmission probability calculations. The introduction of these scaling factors fulfils two purposes: to account for (a) time-reversal symmetry breaking in charge transport experiments, and (b) the presence of the CISS effect, *i.e.*, spin selectivity, which leads to different electrical responses of the system in dependence of the polarization of the incoming electrons from the magnetic tip. A typical transmission function is shown in Fig. 4a for the case where fourteen carbon atoms belonging to the basal plane of NG 1 are assumed to have contact with the bottom electrode, while only one carbon atom is contacted on the top of the molecule (for other number of contacts, see the Fig. S47 in the SI). The choice on the number of carbon atoms in contact with the metallic substrate was based on the performed DFT calculations, by considering atoms with a vertical distance of the order of 3 Å and 4 Å. The transmission function displays the typical peaks at the position of molecular eigenstates, where the broadening of peaks is controlled by the couplings to the electrode. Notice that smaller *β*-values (in this case for the spin-up channel) induce a smaller gap and thus a larger overlap of transmission resonances within the HOMO–LUMO gap. This results in a larger transmission when compared to the spin-down channel. This effect becomes less prominent, as expected, with decreasing *δ*. The inset of Fig. 4a shows the spin asymmetry factor Δ(*E*) as a function of the incoming electron energy for different values of *δ*. Δ(*E*) shows an oscillating behavior as a function of the energy outside the HOMO–LUMO gap.^35^ Notice also that the spin asymmetry within the HOMO–LUMO gap is nonzero and increases with increasing *δ*. In Fig. 4b, we show a voltage-dependent integrated asymmetry factor Δ_V_(*V*) as a function of applied bias *V*. This quantity is defined as Δ_V_(*V*) = 100 × (*I*_↑_(*V*) − *I*_↓_(*V*))/(*I*_↑_(*V*) + *I*_↓_(*V*)), with 
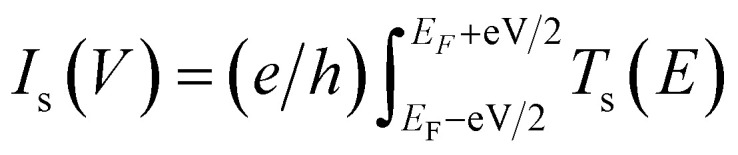
 being the Landauer electrical current at zero temperature. Δ_V_(*V*) is closer, in its definition, to the spin polarization of the current measured experimentally. For larger values of *δ*, Δ_V_(*V*) adopts weaker voltage-dependent values, which is in qualitative agreement with the experimental results. These results suggest, despite the approximations made to simplify the transport model, that non-resonant tunneling within the HOMO–LUMO gap is largely determining the large spin polarization as well as its nearly constant value. At larger voltages, molecular resonances start to contribute, but in this case a full non-equilibrium transport calculation will be needed, making the transmission function voltage dependent.

## Conclusions

In summary, we have demonstrated that lateral extended helical NGs exhibit a clear CISS effect. Thanks to the lateral π-extension, the targeted helical NGs present inherent chirality and intensive chiroptical responses, thus serving as an efficient molecular platform for spin-selective transport. Both mc-AFM and MR measurements demonstrate pronounced spin polarization behavior, highlighting that lateral extended helical NGs can be exploited as novel quantum materials for potential applications based on the CISS effect. This work presents an effective lateral structure regulation strategy, advancing the design of helicene-based NGs with robust spin polarization and large spin-polarized currents.

## Author contributions

W. Niu, Y. Sang, G. Cuniberti and S. Parkin supervised the project. W. Niu, F. Wu and H. Shi synthesized and characterized the compounds. C. Fang, J. Deka performed the MR measurements. L. Tang performed the mc-AFM measurements. E. Unsal, Y. Fu, A. Dianat, and R. Gutierrez carried out the DFT calculations and the spin-dependent transport calculations. F. Liu and A. Popov measured and refined the single crystal structures. H. Komber measured and analysed the NMR spectra. W. Niu, C. Fang, R. Gutierrez and Y. Sang co-wrote the paper with contributions from all co-authors.

## Conflicts of interest

There are no conflicts to declare.

## Supplementary Material

SC-OLF-D5SC03887A-s001

SC-OLF-D5SC03887A-s002

## Data Availability

CCDC 2412066 contains the supplementary crystallographic data for this paper.^36^ Experimental and computational data can be found in the SI. The cif-files of the crystal structures can be found alongside the SI. See DOI: https://doi.org/10.1039/d5sc03887a.

## References

[cit1] Bloom B. P., Paltiel Y., Naaman R., Waldeck D. H. (2024). Chem. Rev..

[cit2] Naaman R., Paltiel Y., Waldeck D. H. (2020). Acc. Chem. Res..

[cit3] Naaman R., Paltiel Y., Waldeck D. H. (2019). Nat. Rev. Chem..

[cit4] Ray K., Ananthavel S. P., Waldeck D. H., Naaman R. (1999). Science.

[cit5] Zhang D.-Y., Sang Y., Das T. K., Guan Z., Zhong N., Duan C.-G., Wang W., Fransson J., Naaman R., Yang H.-B. (2023). J. Am. Chem. Soc..

[cit6] Eckvahl H. J., Tcyrulnikov N. A., Chiesa A., Bradley J. M., Young R. M., Carretta S., Krzyaniak M. D., Wasielewski M. R. (2023). Science.

[cit7] Aizawa H., Sato T., Maki-Yonekura S., Yonekura K., Takaba K., Hamaguchi T., Minato T., Yamamoto H. M. (2023). Nat. Commun..

[cit8] Mondal A. K., Preuss M. D., Ślęczkowski M. L., Das T. K., Vantomme G., Meijer E. W., Naaman R. (2021). J. Am. Chem. Soc..

[cit9] Xu Y., Mi W. (2023). Mater. Horiz..

[cit10] Han X., Jiang C., Hou B., Liu Y., Cui Y. (2024). J. Am. Chem. Soc..

[cit11] Qian Q., Ren H., Zhou J., Wan Z., Zhou J., Yan X., Cai J., Wang P., Li B., Sofer Z., Li B., Duan X., Pan X., Huang Y., Duan X. (2022). Nature.

[cit12] Safari M. R., Matthes F., Schneider C. M., Ernst K.-H., Bürgler D. E. (2024). Small.

[cit13] Giaconi N., Poggini L., Lupi M., Briganti M., Kumar A., Das T. K., Sorrentino A. L., Viglianisi C., Menichetti S., Naaman R., Sessoli R., Mannini M. (2023). ACS Nano.

[cit14] Rodríguez R., Naranjo C., Kumar A., Matozzo P., Das T. K., Zhu Q., Vanthuyne N., Gómez R., Naaman R., Sánchez L., Crassous J. (2022). J. Am. Chem. Soc..

[cit15] Rodríguez R., Naranjo C., Kumar A., Dhbaibi K., Matozzo P., Camerel F., Vanthuyne N., Gómez R., Naaman R., Sánchez L., Crassous J. (2023). Chem.–Eur. J..

[cit16] Chiesa A., Privitera A., Macaluso E., Mannini M., Bittl R., Naaman R., Wasielewski M. R., Sessoli R., Carretta S. (2023). Adv. Mater..

[cit17] Kim Y.-H., Zhai Y., Lu H., Pan X., Xiao C., Gaulding E. A., Harvey S. P., Berry J. J., Vardeny Z. V., Luther J. M., Beard M. C. (2021). Science.

[cit18] Shang Z., Liu T., Yang Q., Cui S., Xu K., Zhang Y., Deng J., Zhai T., Wang X. (2022). Small.

[cit19] Mishra S., Mondal A. K., Pal S., Das T. K., Smolinsky E. Z. B., Siligardi G., Naaman R. (2020). J. Phys. Chem. C.

[cit20] Göhler B., Hamelbeck V., Markus T. Z., Kettner M., Hanne G. F., Vager Z., Naaman R., Zacharias H. (2011). Science.

[cit21] Metzger T. S., Batchu H., Kumar A., Fedotov D. A., Goren N., Bhowmick D. K., Shioukhi I., Yochelis S., Schapiro I., Naaman R., Gidron O., Paltiel Y. (2023). J. Am. Chem. Soc..

[cit22] Izquierdo-García P., Fernández-García J. M., Medina Rivero S., Šámal M., Rybáček J., Bednárová L., Ramírez-Barroso S., Ramírez F. J., Rodríguez R., Perles J., García-Fresnadillo D., Crassous J., Casado J., Stará I. G., Martín N. (2023). J. Am. Chem. Soc..

[cit23] Tian X., Shoyama K., Mahlmeister B., Brust F., Stolte M., Würthner F. (2023). J. Am. Chem. Soc..

[cit24] Niu W., Fu Y., Deng Q., Qiu Z.-L., Liu F., Popov A. A., Komber H., Ma J., Feng X. (2024). Angew. Chem., Int. Ed..

[cit25] Niu W., Fu Y., Qiu Z.-L., Schürmann C. J., Obermann S., Liu F., Popov A. A., Komber H., Ma J., Feng X. (2023). J. Am. Chem. Soc..

[cit26] Shen Y.-J., Yao N.-T., Diao L.-N., Yang Y., Chen X.-L., Gong H.-Y. (2023). Angew. Chem., Int. Ed..

[cit27] Chen Y., Lin C., Luo Z., Yin Z., Shi H., Zhu Y., Wang J. (2021). Angew. Chem., Int. Ed..

[cit28] Cruz C. M., Castro-Fernández S., Maçôas E., Cuerva J. M., Campaña A. G. (2018). Angew. Chem., Int. Ed..

[cit29] Morita F., Kishida Y., Sato Y., Sugiyama H., Abekura M., Nogami J., Toriumi N., Nagashima Y., Kinoshita T., Fukuhara G., Uchiyama M., Uekusa H., Tanaka K. (2024). Nat. Synth..

[cit30] Shen Y.-J., Peng L.-J., Diao L.-N., Yao N.-T., Chen W.-K., Yang Y., Qiu M., Zhu W.-X., Li X., Wang X.-Y., Gong H.-Y. (2024). Org. Lett..

[cit31] Zhu K.-L., Li Z.-A., Liang J., Zou K.-L., Shen Y.-J., Gong H.-Y. (2024). Angew. Chem., Int. Ed..

[cit32] Jiang H., Čavlović D., Jiang Q., Ng F., Bao S. T., Telford E. J., Steigerwald M. L., Roy X., Nuckolls C., McNeill J. M. (2025). J. Am. Chem. Soc..

[cit33] Varela S., Gutierrez R., Cuniberti G., Medina E., Mujica V. (2024). J. Chem. Phys..

[cit34] Liu T., Weiss P. S. (2023). ACS Nano.

[cit35] Gutierrez R., Díaz E., Gaul C., Brumme T., Domínguez-Adame F., Cuniberti G. (2013). J. Phys. Chem. C.

[cit36] Niu W. Fang C. , Tang L., Unsal E., Fu Y., Deka J., Liu F., Popov A. A., Wu F., Shi H., Komber H., Dianat A., Gutierrez R., Ma J., Sang Y., Cuniberti G. and Parkin S. S. P., CCDC 2412066 Experimental Crystal Structure Determination, 2025, 10.5517/ccdc.csd.cc2lyyl3

