# Supplementary material for: Lateral π-extended helical nanographenes with large spin polarization
Source: Chem Sci. 2025 Aug 13;16(45):21446–53. doi: 10.1039/d5sc03887a (PMC12365924; doi:10.1039/d5sc03887a)
Supplement: SC-016-D5SC03887A-s001 [file SC-016-D5SC03887A-s001.pdf]

Supplementary Information (SI) for Chemical Science.
This journal is © The Royal Society of Chemistry 2025

Supporting Information

Lateral π-Extended Helical Nanographenes with Large Spin Polarization

Wenhui Niu,[a],[b],[l],* Chi Fang,[a],[l] Likun Tang,[c],[l] Elif Unsal,[d] Yubin Fu,[a],[b] Jitul Deka,[a] Fupin Liu,[e],[f] Alexey A. Popov,[e] Fupeng Wu,[g] Huanhuan Shi,[h] Hartmut Komber,[i] Arezoo Dianat,[d] Rafael Gutierrez,[d] Ji Ma,[a],[b],[j] Yutao Sang,[c],* Gianaurelio Cuniberti,[d],[k],* Stuart S. P. Parkin[a],*

[a] Dr. W. Niu, Dr. C. Fang, Dr. Y. Fu, J. Deka, Dr. J. Ma, Prof. Dr. S. Parkin
Max Planck Institute of Microstructure Physics, Weinberg 2, 06120 Halle, Germany
E-mail: wenhui.niu@mpi-halle.mpg.de; stuart.parkin@mpi-halle.mpg.de

[b] Dr. W. Niu, Dr. Y. Fu, Dr. J. Ma
Center for Advancing Electronics Dresden (cfaed) & Faculty of Chemistry and Food Chemistry, Technische Universität Dresden, Mommsenstraße 4, 01062 Dresden, Germany

[c] L. Tang, Dr. Y. Sang
State Key Laboratory of Molecular Engineering of Polymers, Shanghai Key Laboratory of Metasurfaces for Light Manipulation, Department of Macromolecular Science, Fudan University, 200438 Shanghai, P. R. China
Email: sangyt@fudan.edu.cn

[d] E. Unsal, Dr. A. Dianat, Dr. R. Gutierrez, Prof. Dr. G. Cuniberti
Institute for Materials Science and Max Bergmann Center of Biomaterials, TU Dresden, 01062 Dresden, Germany
Email: gianaurelio.cuniberti@tu-dresden.de

[e] Dr. F. Liu, Dr. A. A. Popov
Leibniz Institute for Solid State and Materials Research (IFW Dresden), Helmholtzstraße 20, 01069, Dresden, Germany

[f] Dr. F. Liu
Jiangsu Key Laboratory of New Power Batteries, Jiangsu Collaborative Innovation Center of Biomedical Functional Materials, School of Chemistry and Materials Science, Nanjing Normal University, 210023 Nanjing, P. R. China

[g] Dr. F. Wu
Max Planck Institute Chemical Physics of Solids, Nöthnitzer Straße 40, 01187 Dresden, Germany

[h] Dr. H. Shi
Karlsruhe Institute of Technology, Institute for Quantum Materials and Technologies, Kaiserstraße 12, 76131 Karlsruhe, Germany

[i] Dr. H. Komber
Leibniz-Institut für Polymerforschung Dresden e. V., Hohe Straße 6, 01069 Dresden, Germany

[j] Dr. J. Ma
College of Materials Science and Opto-Electronic Technology & Center of Materials Science and Optoelectronics Engineering, University of Chinese Academy of Science, 100049 Beijing, P. R. China

[k] Prof. Dr. G. Cuniberti
Dresden Center for Computational Materials Science (DCMS), TU Dresden, 01062 Dresden, Germany

[l] These authors contributed equally to this work.

Content

1 General methods and materials S2

2 Detailed synthetic procedure of **1** and **2** and their structural characterizations S3

3 Optical properties and DFT calculations S26

4 mc-AFM measurements S29

5 Magnetoresistance measurements S31

6 Computational methodology and spin transport calculations S32

7 References S35

1. General methods and materials

All the reagents were obtained from Sigma Aldrich, TCI, abcr, BLD Pharm, Strem. All these chemicals were used as received without further purification. All reactions dealing with air- or moisture-sensitive compounds were carried out in a dry reaction vessel under Ar atmosphere. Anhydrous dichloromethane and tetrahydrofuran were obtained from MBRAUN MB-SPS-5 solvent purification system. All the sensitive reactions were performed using standard vacuum-line and Schlenk techniques.

Thin layer chromatography (TLC) was performed on silica-coated aluminium sheets with a fluorescence indicator (TLC silica gel 60 F254, purchased from Merck KGaA). Column chromatography was performed on silica (SiO_2, particle size 0.063-0.200 mm, purchased from VWR).

NMR spectra were recorded on a Bruker AV-II 300 spectrometer operating at 300 MHz for ^{1}H and at 75 MHz for ^{13}C at room temperature (23°C) and on a Bruker Avance III 500 spectrometer operating at 500.13 MHz for ^{1}H and at 125.77 MHz for ^{13}C at 30°C (unless otherwise stated). The 1D and 2D NMR spectra were recorded using the standard Bruker pulse programs. Chemical shifts (δ) are reported in ppm. The following abbreviations are used to describe peak patterns as appropriate: s = singlet, d = doublet, t = triplet, q = quartet, and m = multiplet.

The mass spectrometry analysis was performed on a Bruker Autoflex Speed MALDI TOF MS (Bruker Daltonics, Bremen, Germany) using *trans*-2-[3-(4-*tert*-butylphenyl)-2-methyl-2-propenylidene]malononitrile as matrix.

UV-visible spectra were measured on an Agilent Cary 5000 UV-vis-NIR spectrophotometer by using 10 mm optical-path quartz cell at room temperature. Fluorescence spectra were recorded at room temperature on a PerkinElmer Fluorescence Spectrometer LS 55 using a 10 mm fluorescence quartz cell. Absolute quantum yields were measured at room temperature on a C11347-01 absolute PL Quantum Yield Spectrometer from Hamamatsu Photonics company using a 10 mm fluorescence quartz cell.

High-performance liquid chromatography (HPLC) was carried out with a Shimadzu FCV-20AH2 system and Whelk-O (250 mm×21.1 mm) column.

Magneto-conductive atomic force microscopy (mc-AFM) results were performed using the contact mode (Oxford) under ambient conditions. Substrate surfaces were prepared by sputtering a 100 nm gold (Au) on top of a silicon wafer. All surfaces were first cleaned by immersing in boiling acetone and then in ethanol for 10 min, followed by a UV-ozone cleaning for 15 min. Samples for mc-AFM measurements were prepared by spin-coating the diluted solutions of NGs on the surface. The thin films were then annealed under 60°C for 1 min before measurement. Current-voltage (*I*-*V*) spectroscopy measurements were recorded by performing voltage ramps with the tip in contact with the surface. Co-Cr–coated tips (ASYMFM, Oxford, with spring constant of 2.8 N/m) were pre-magnetized by a strong permanent magnet (~0.5 T) for >30 min and then used for the scan immediately. If the measurement time was longer than 60 min, the tip was placed back to the same pole of the magnetic field and magnetized for 30 min again. *I*-*V* curves were acquired by ramping the voltage from −2.5 to +2.5 V, with a frequency of 0.5 Hz. At least 30 *I*–*V* traces were recorded and averaged for each location and magnetic field orientation. All the recorded traces were provided in the supporting information. For each *I*–*V* measurement, the tip was placed in a new location, and movement between points was done with the tip lifted from the surface to prevent damage to the sample. At least three locations were measured for each sample.

2. Detailed synthetic procedure of 1 and 2 and their structural characterization

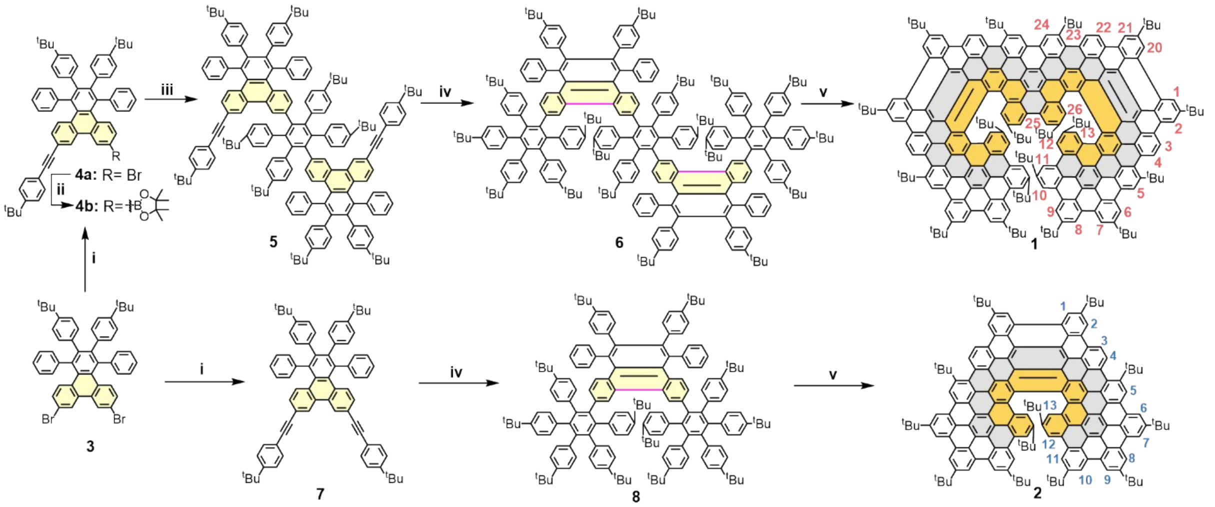

*Scheme S1. (a) Synthetic route to lateral extended undecabenzo[7]helicenes (**1** and **2**). (i) 4-(tert-butyl)phenylacetylene, $Pd(PPh_3)_2Cl_2$, CuI, THF, Et_3N, 80 °C, 24 h, 20% for **4a**; 10% for 7; (ii) Pd(dppf)Cl_2, KOAc, bis(pinacolato)diboron, dioxane, 85 °C, 18 h, 61% for **4b**; (iii) $Pd(PPh_3)_4$, K_2CO_3, dioxane, H_2O, 95 °C, 24 h, 49 %; (iv) tetrakis(4-(tert-butyl)phenyl)cyclopenta-2,4-dien-1-one, Ph2O, 270 °C, 24 h, 80 % for **6**, and 84% for **8**; (v) DDQ, DCM, TfOH, 0 °C, 40 min, 41 % for **1**, and 73 % for **2**.*

The synthesis of compound **3** is following reported work.[1]

Synthesis of 7-bromo-2,3-bis(4-(*tert*-butyl)phenyl)-10-((4-(*tert*-butyl)phenyl)ethynyl)-1,4-diphenyltriphenylene (4a) and 2,3-bis(4-(*tert*-butyl)phenyl)-7,10-bis((4-(*tert*-butyl)phenyl)ethynyl)-1,4-diphenyltriphenylene (7)

To a degassed suspension of compound **3** (3.0 g, 3.74 mmol) and copper(I) iodide (142 mg, 0.75 mmol) in mixture solution of triethylamine (8 mL) and THF (80 mL) were added 4-(*tert*-butyl)phenylacetylene (769 mg, 4.86 mmol) and $Pd(PPh_3)_2Cl_2$ (131 mg, 0.19 mmol). After stirring at 80 °C for 24 h, the solvent was cooled down to room temperature. The reaction mixture was washed by saturated aqueous solution of ammonium chloride, and then extracted by ethyl acetate. Subsequently, the organic layer was collected, dried over magnesium sulfate, and evaporated. Purification by silica gel column chromatography (eluent: iso-hexane/DCM = 10/1) yielded the white solid **4a** (0.66 g, 20%) and yellow solid **7** (350 mg, 10%).

4a: ^{1}H NMR (300 MHz, CD_2Cl_2) δ 8.58 (s, 1H), 8.53 (s, 1H), 7.54 – 7.39 (m, 6H), 7.10 (m, 12H), 6.90 (dd, J = 8.4, 1.4 Hz, 4H), 6.59 (dd, J = 8.4, 1.5 Hz, 4H), 1.34 (s, 9H), 1.17 (m, 18H). ^{13}C NMR (75 MHz, CD_2Cl_2) δ 152.50, 148.84, 143.08, 142.21, 142.10, 137.92, 137.67, 133.24, 132.65, 132.14, 131.87, 131.49, 131.12, 130.83, 130.49, 129.16, 129.10, 128.60, 128.57, 126.93, 126.84, 126.52, 126.08, 123.87, 121.81, 121.18, 120.56, 90.91, 89.24, 34.63, 31.57, 31.48.

HR-MALDI (m/z): calculated for $C_{62}H_{55}Br$ $[M]^+$ 880.3484; found, 880.3691.

7: ^{1}H NMR (300 MHz, CD_2Cl_2) δ 8.64 (s, 2H), 7.55 – 7.48 (m, 6H), 7.43 – 7.39 (m, 4H), 7.16 – 7.07 (m, 12H), 6.90 (d, J = 8.4 Hz, 4H), 6.60 (d, J = 8.4 Hz, 4H), 1.34 (s, 18H), 1.27 (s, 18H).

^{13}C NMR (75 MHz, CD_2Cl_2) δ 152.46, 148.82, 143.19, 142.13, 137.87, 132.68, 131.88, 131.53, 130.48, 128.82, 128.55, 126.94, 126.08, 123.87, 121.75, 120.65, 90.80, 89.42, 31.57, 31.48.

HR-MALDI (m/z): calculated for $C_{74}H_{68}$ $[M]^+$ 956.5321; found, 956.5350.

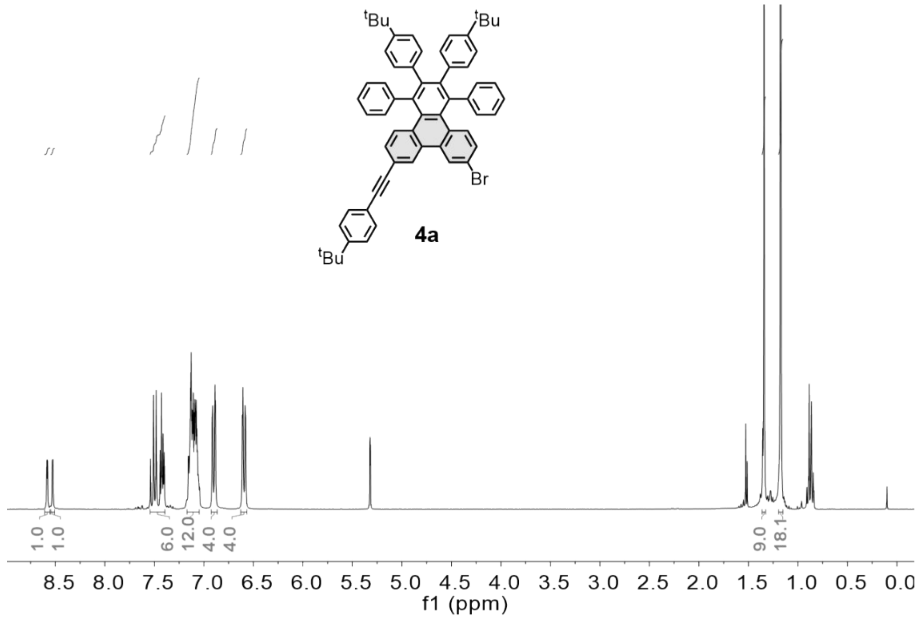

Figure S1. ^{1}H NMR spectrum (300 MHz, CD_2Cl_2) of ***4a****.*

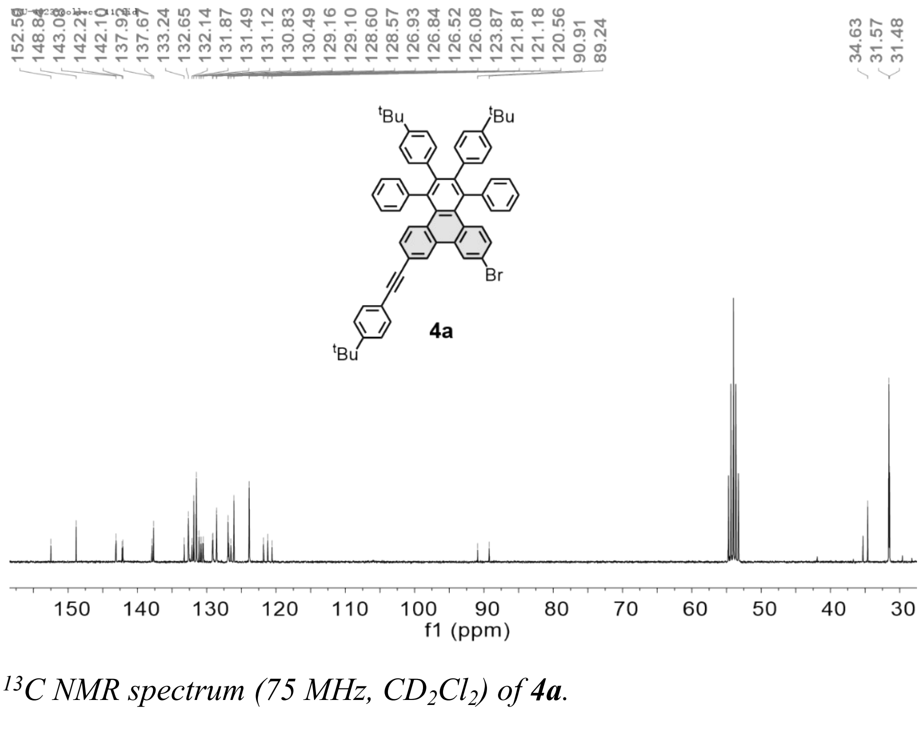

Figure S2. ^{13}C NMR spectrum (75 MHz, CD_2Cl_2) of ***4a****.*

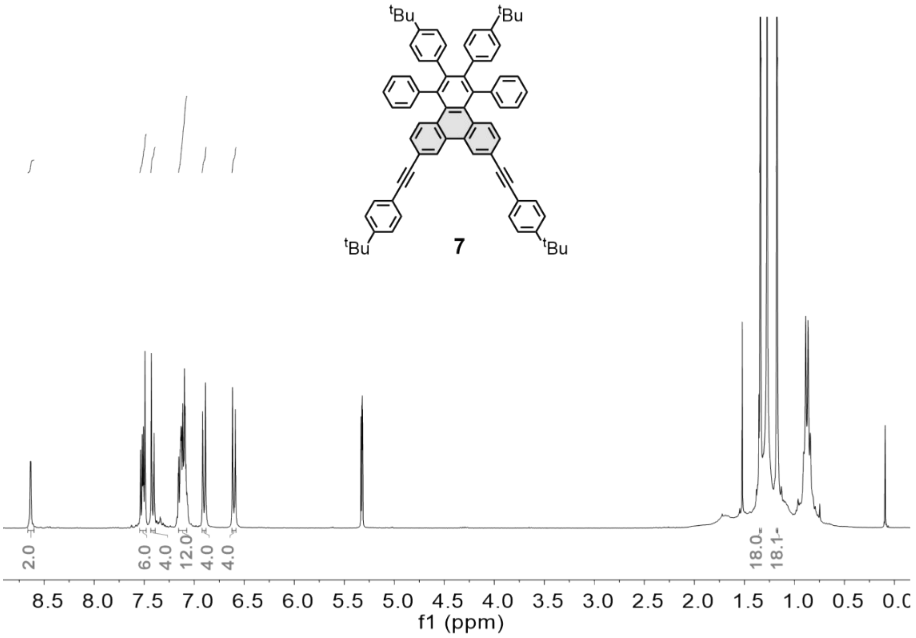

Figure S3. ^{1}H NMR spectrum (300 MHz, CD_2Cl_2) of 7.

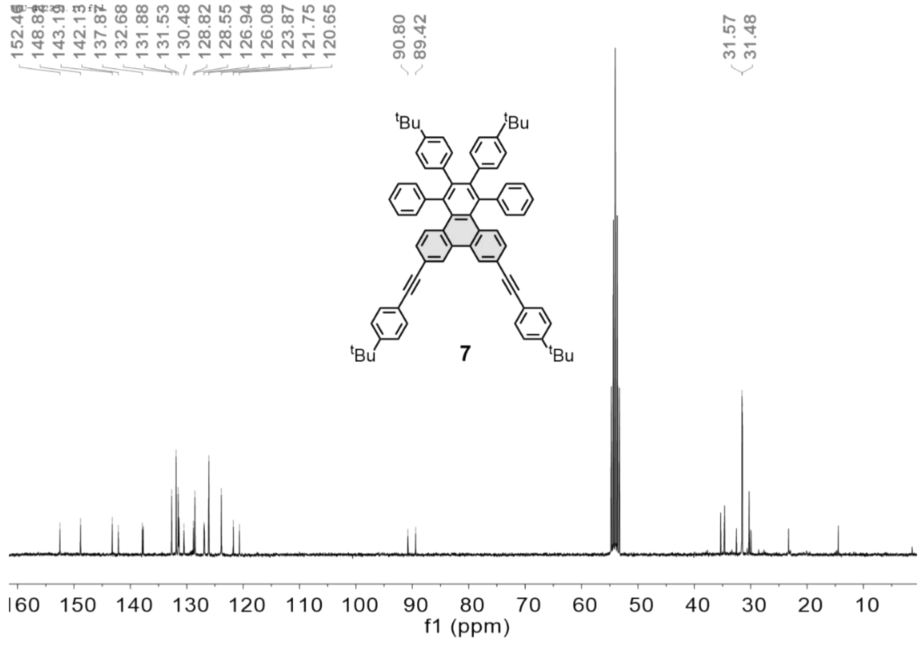

Figure S4. ^{13}C NMR spectrum (75 MHz, CD_2Cl_2) of 7.

Synthesis of 2-(6,7-bis(4-(*tert*-butyl)phenyl)-11-((4-(*tert*-butyl)phenyl)ethynyl)-5,8-diphenyltriphenylen-2-yl)-4,4,5,5-tetramethyl-1,3,2-dioxaborolane (4b)

An oven-dried Schlenk tube was charged with Pd(dppf)Cl_2 (19 mg, 23 µmol), bis(pinacolato)diboron (462 mg, 1.82 mmol) and KOAc (89 mg, 0.91 mmol), **4a** (200 mg, 0.23 mmol). Then evacuated and backfilled with Ar (this sequence was carried out twice). 1,4-Dioxane (7 mL) was added via syringe, through the septum. The reaction mixture was stirred at 85 °C for 18 h. Then the reaction mixture was filtered through a thin pad of celite (eluting with ethyl acetate) and the filtrate was concentrated under reduced pressure. The residue was

purified by silica column chromatography (eluent: iso-hexane/DCM= 3:1) to afford **4b** (128 mg, 61%) as a white solid.

^{1}H NMR (300 MHz, CD_2Cl_2) δ 8.88 (s, 1H), 8.72 (s, 1H), 7.56 – 7.48 (m, 4H), 7.44 – 7.35 (m, 3H), 7.15 – 7.05 (m, 11H), 6.90 (d, J = 8.3 Hz, 4H), 6.63 – 6.56 (d, J = 8.3 Hz, 4H), 1.37 (s, 12H), 1.34 (s, 9H), 1.17 (s, 18H). ^{13}C NMR (75 MHz, CD_2Cl_2) δ 152.40, 148.78, 143.34, 143.24, 142.12, 141.98, 138.01, 137.77, 137.74, 133.91, 132.69, 132.07, 131.87, 131.52, 131.19, 130.51, 130.38, 129.63, 128.56, 128.41, 126.79, 126.07, 123.84, 121.59, 120.69, 90.66, 89.54, 84.54, 35.29, 34.62, 31.56, 31.47, 25.29.

HR-MALDI (m/z): calculated for $C_{68}H_{67}BO_2$ $[M]^+$ 926.5234; found, 926.5190.

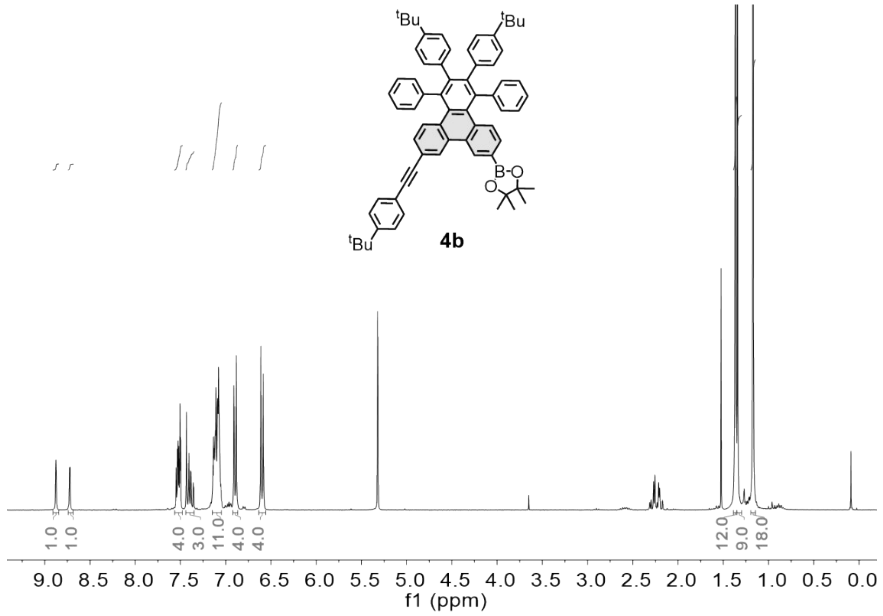

*Figure S5. ^{1}H NMR spectrum (300 MHz, CD_2Cl_2) of **4b**.*

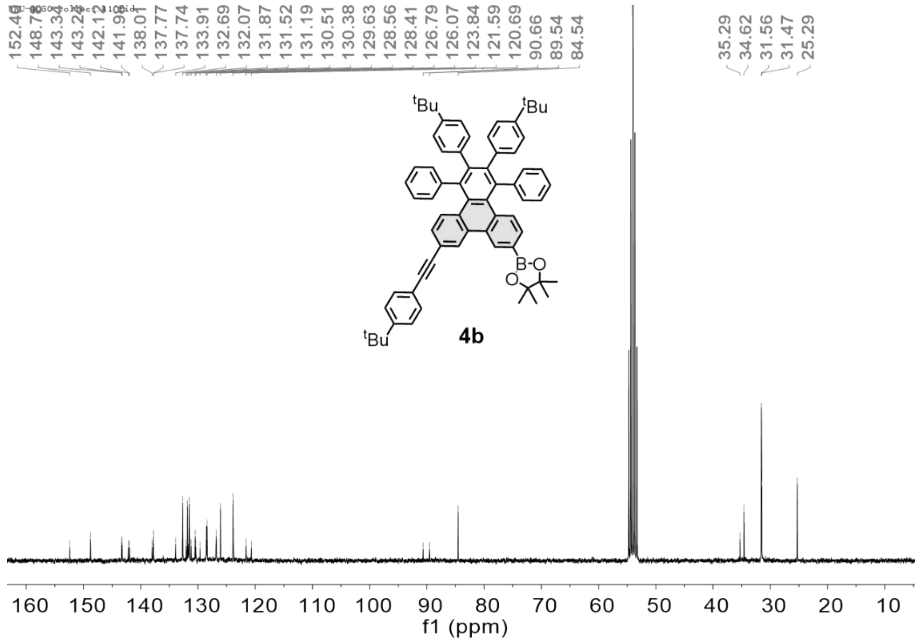

*Figure S6. ^{13}C NMR spectrum (75 MHz, CD_2Cl_2) of **4b**.*

Synthesis of 4,4''-di-*tert*-butyl-4',5'-bis(4-(*tert*-butyl)phenyl)-3',6'-diiodo-1,1':2',1''-terphenyl

See reference [2] for the detailed procedure.

Synthesis of 10,10'-(4,4''-di-*tert*-butyl-4',5'-bis(4-(*tert*-butyl)phenyl)-[1,1':2',1''-terphenyl]-3',6'-diyl)bis(2,3-bis(4-(tert-butyl)phenyl)-7-((4-(*tert*-butyl)phenyl)ethynyl)-1,4-diphenyltriphenylene) (5)

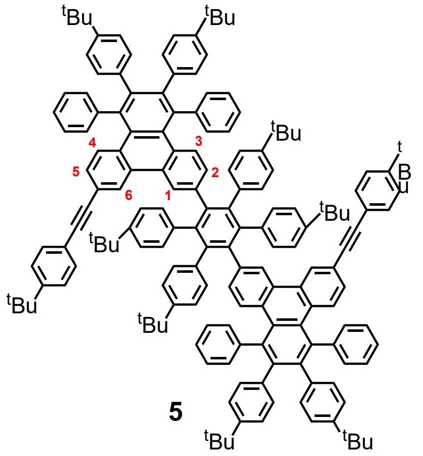

5

A flask was charged with **4b** (189 mg, 0.20 mmol), 4,4''-di-*tert*-butyl-4',5'-bis(4-(*tert*-butyl)phenyl)-3',6'-diiodo-1,1':2',1''-terphenyl (70 mg, 82 μmol) and K_2CO_3 (227 mg, 1.6 mmol) along with 5 mL of dioxane and 0.8 mL water. The mixture was degassed by Ar bubbling and then $Pd(PPh_3)_4$ (19 mg, 16 μmol) was added. The solution was further degassed by Ar bubbling for another 10 min. Then the reaction was stirred at 100 °C under Ar for 24 h. Afterwards, the reaction mixture was diluted with DCM (50 mL). The mixture was washed three times with water, dried over sodium sulfate, and evaporated. The solid was purified by silica column chromatography (eluent: iso-hexane/DCM=8/1) to afford **5** (89 mg, 49%) as a white solid.

Compound **5** is a mixture of several isomers formed by rotation around single bonds. Both the ^1H and ^{13}C NMR spectra are characterised by a large number of overlapping signals in both the aromatic and aliphatic protons and carbons region. This makes it difficult to list the ^1H and ^{13}C NMR signals in a meaningful way. For this reason, both the overview spectrum and the region of aromatic protons/carbons are shown for reference (Figure S7-8 and S10). The EXSY spectrum shows that exchange processes (rotation around single bonds) take place and contribute to the complexity of the spectra (Figure S9). The HSQC spectrum allows the identification of the aromatic CH carbons (Figure S11).

HR-MALDI (m/z): calculated for $C_{170}H_{162}$ $[M]^+$ 2204.1712; found, 2204.2793.

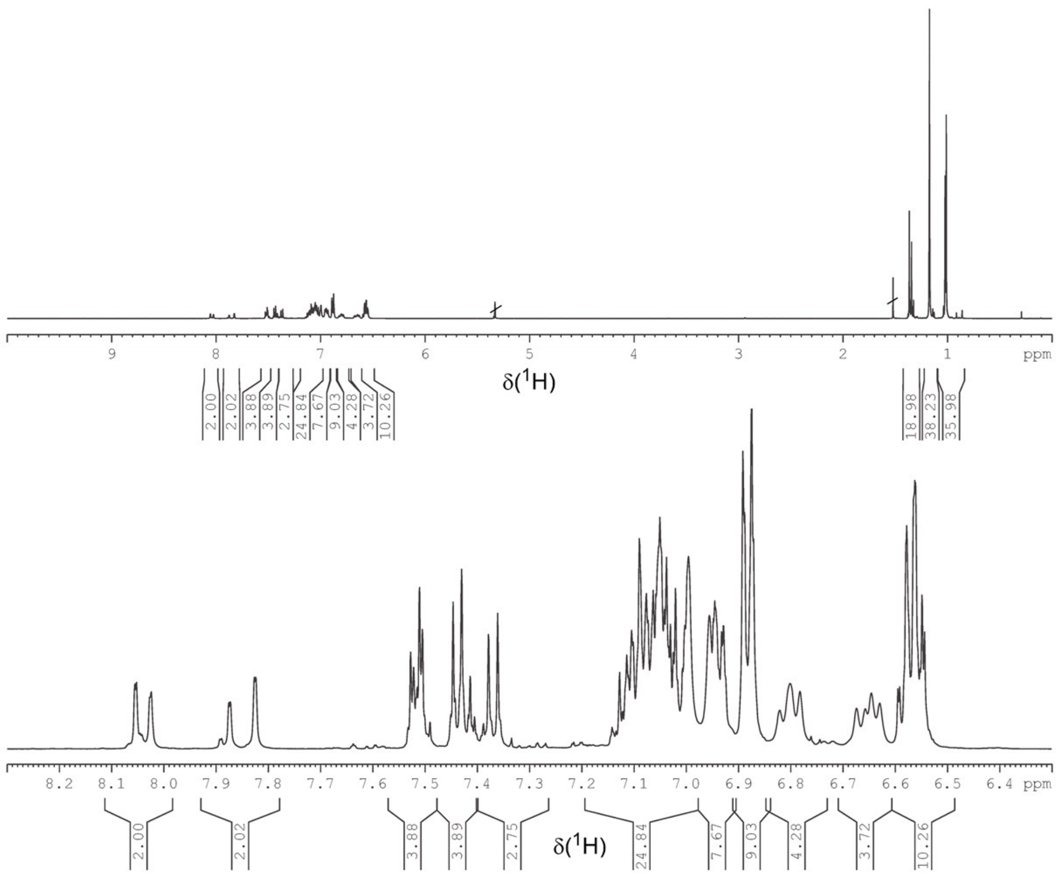

*Figure S7. ¹H NMR spectrum (500 MHz, CD_2Cl_2) of **5** (overview and enlarged region).*

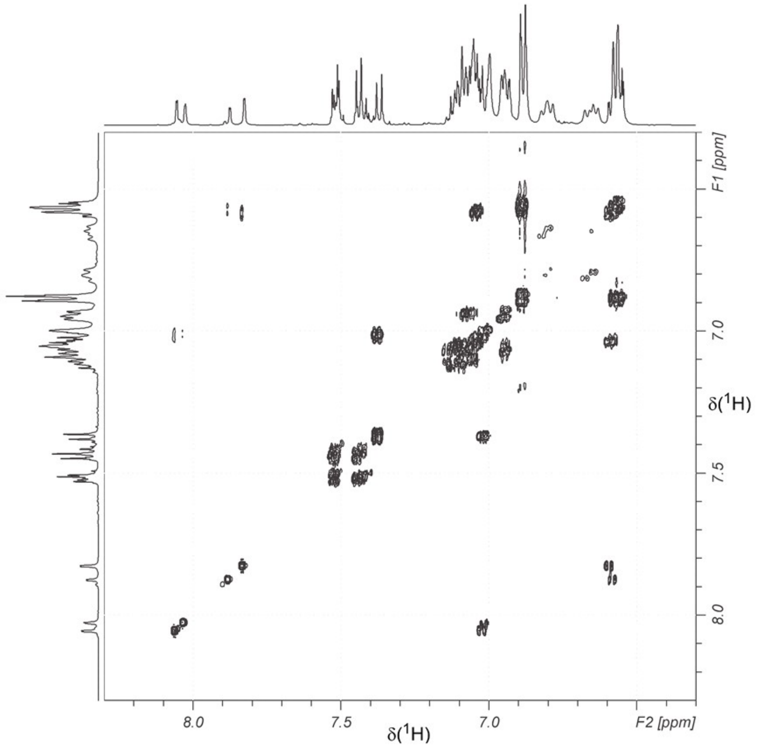

*Figure S8. COSY spectrum of **5** (CD_2Cl_2).*

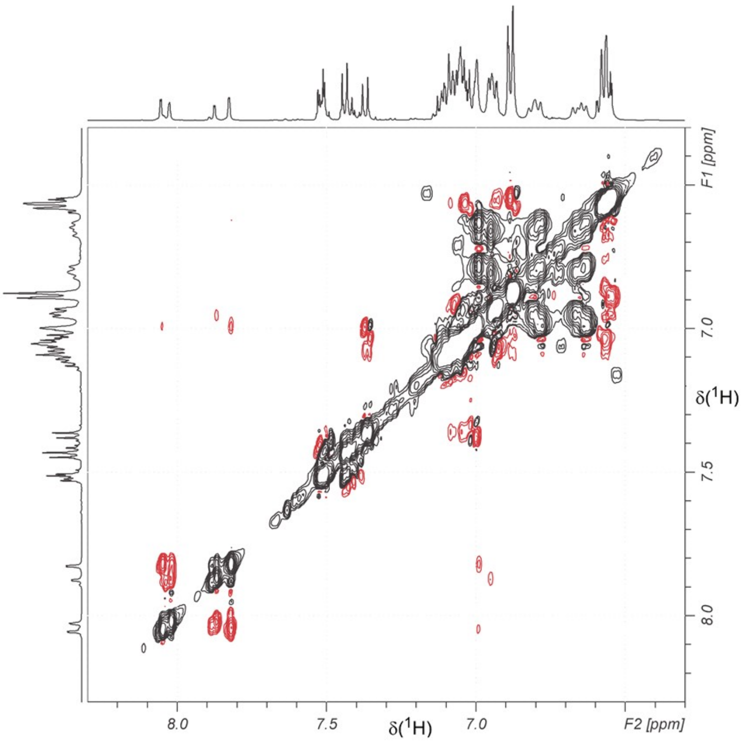

*Figure S9. EXSY (black correlations)/ROESY (red correlations) spectrum of **5** (CD_2Cl_2).*

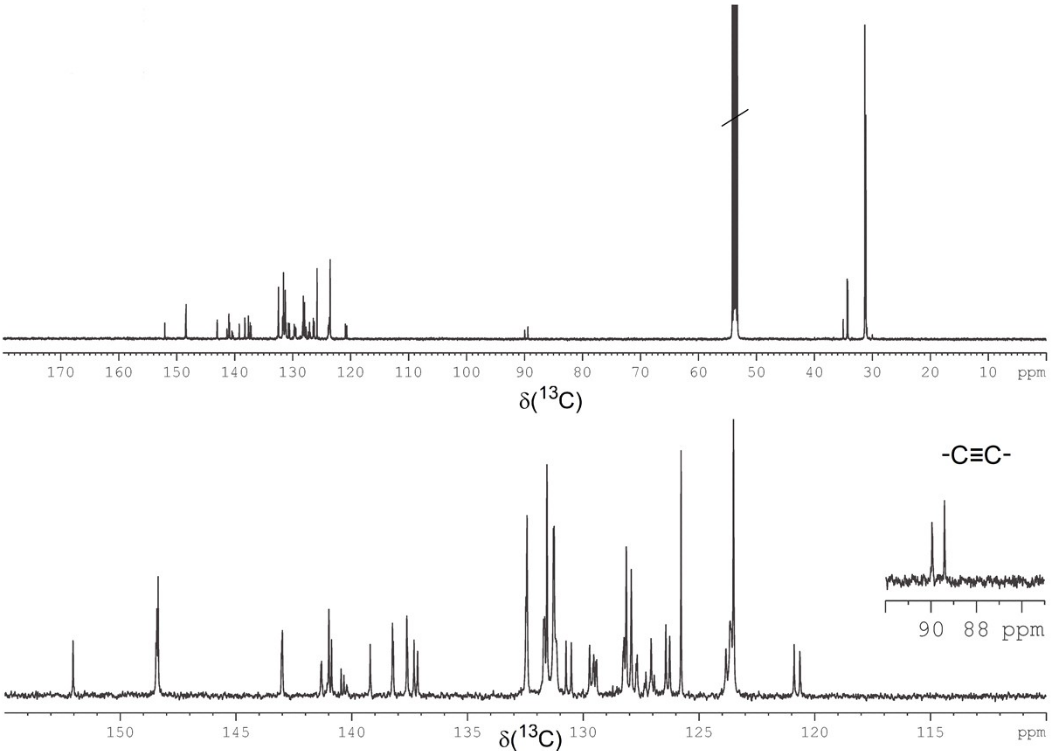

*Figure S10. ^{13}C NMR spectrum (125 MHz, CD_2Cl_2) of **5** (overview and enlarged region).*

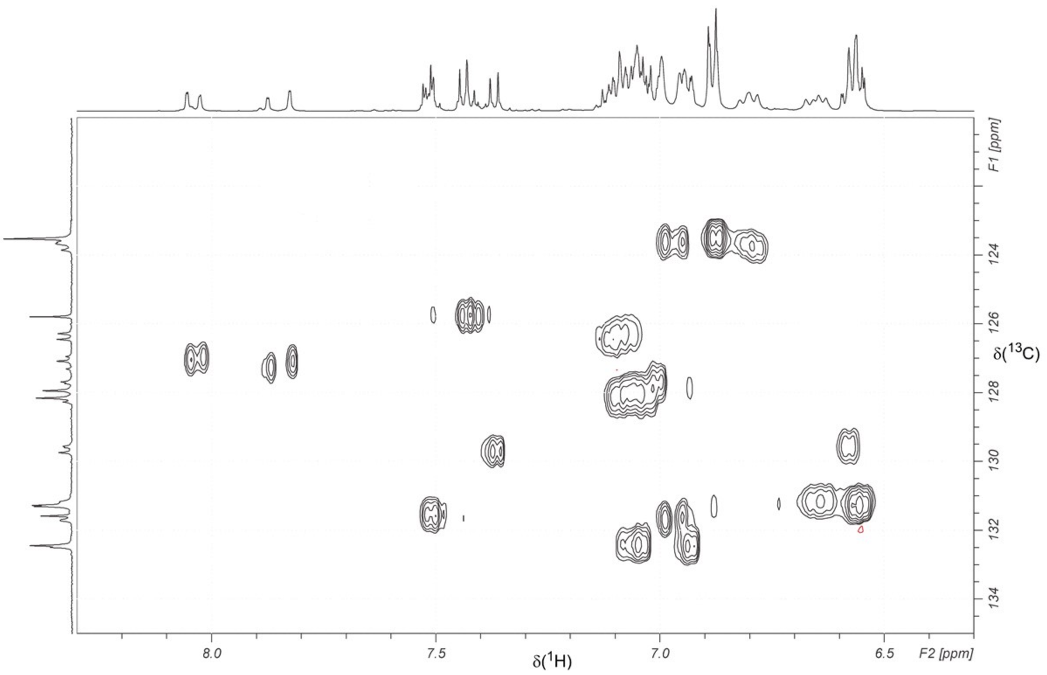

*Figure S11. HSQC spectrum (CD_2Cl_2) of **5** (region of aromatic carbons). The F1 trace is the DEPT135 spectrum.*

Synthesis of 10,10'-(4,4''-di-*tert*-butyl-4',5'-bis(4-(*tert*-butyl)phenyl)-[1,1':2',1''-terphenyl]-3',6'-diyl)bis(2,3-bis(4-(*tert*-butyl)phenyl)-7-(4,4''-di-*tert*-butyl-4',5',6'-tris(4-(tert-butyl)phenyl)-[1,1':2',1''-terphenyl]-3'-yl)-1,4-diphenyltriphenylene) (6) and 2,3-bis(4-(tert-butyl)phenyl)-7,10-bis(4,4''-di-tert-butyl-4',5',6'-tris(4-(tert-butyl)phenyl)-[1,1':2',1''-terphenyl]-3'-yl)-1,4-diphenyltriphenylene (8)

In a 10 mL Schleck tube, a degassed solution of compound **5** (70 mg, 32 μmol) or **7** (52 mg, 54 μmol) and tetrakis(4-(*tert*-butyl)phenyl)cyclopenta-2,4-dien-1-one (100 mg, 0.16 mmol) or (165 mg, 0.27 mmol) in diphenyl ether (0.4 mL) was refluxed for 24 h, and MeOH was added at room temperature. The resulting crude product was collected by filtration and washed by cold methonal twice, yielded the compound **6** (86 mg, 80%) and **8** (96 mg, 84%) as the white solid.

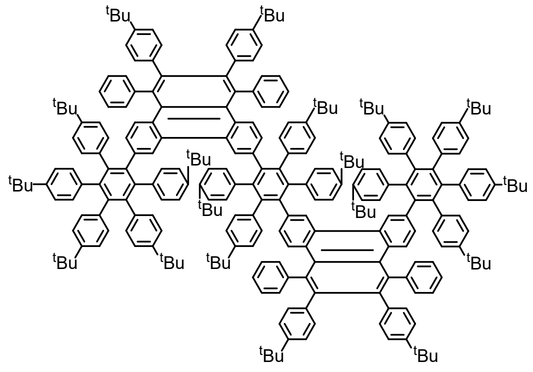

6

Compound **6** is a mixture of several isomers formed by rotation around single bonds. Both the ^{1}H and ^{13}C NMR spectra are characterised by a large number of overlapping signals in both the aromatic and aliphatic protons and carbons region. This makes it difficult to list the ^{1}H and ^{13}C NMR signals in a meaningful way. For this reason, both the overview spectrum and and the region of aromatic protons / carbons are shown for reference (Figure S12-13 and S15). The

EXSY spectrum shows that exchange processes (rotation around single bonds) take place and contribute to the complexity of the spectra (Figure S14). The HSQC spectrum allows the identification of the aromatic CH carbons (Figure S16).

HR-MS MALDI (m/z): calculated for $C_{258}H_{266}$ $[M]^+$ 3366.0882; found, 3366.0752.

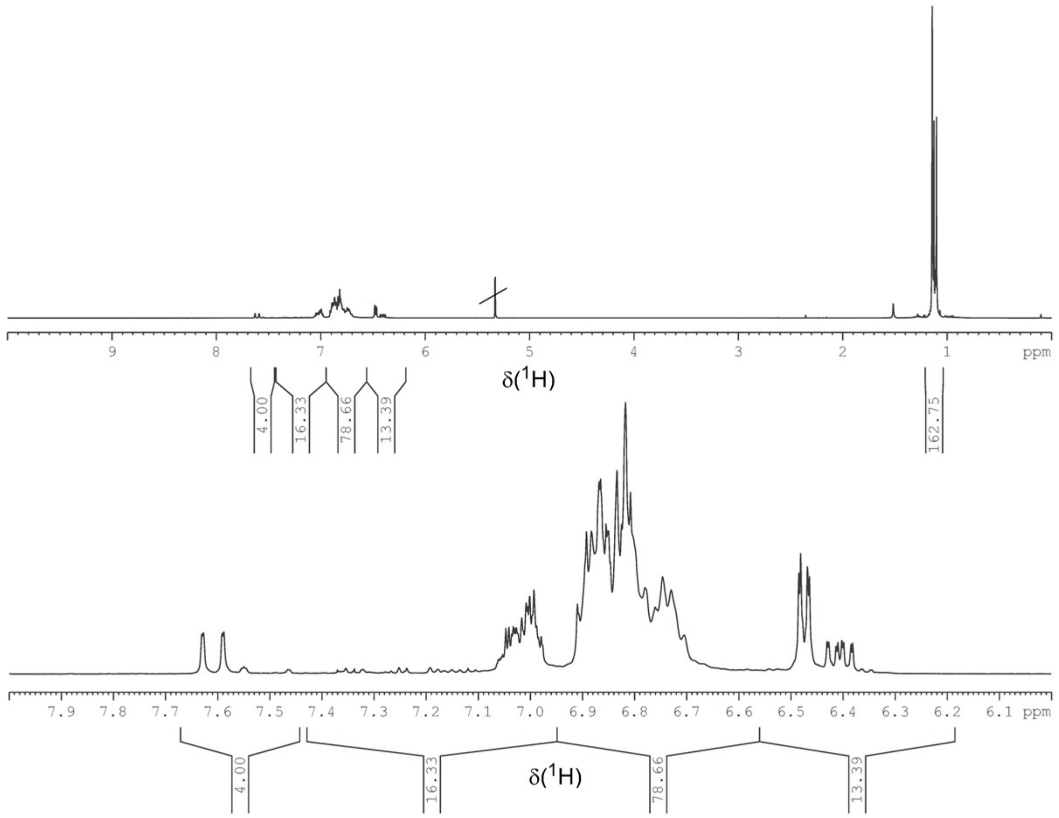

Figure S12. ¹H NMR spectrum (500 MHz, CD_2Cl_2) of ***6*** *(overview and enlarged region).*

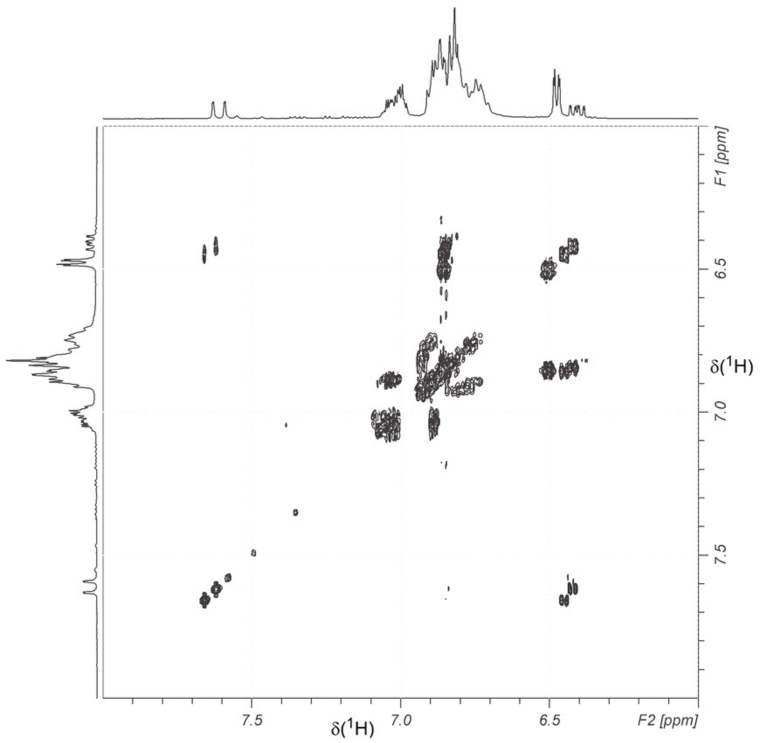

Figure S13. COSY spectrum of ***6*** *(CD_2Cl_2).*

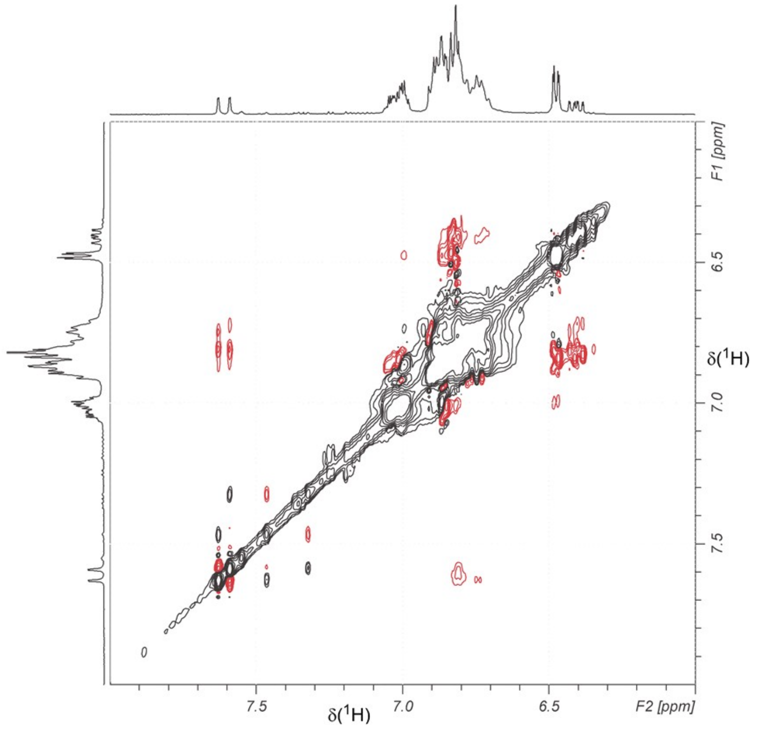

Figure S14. EXSY (black correlations)/ROESY (red correlations) spectrum of ***6*** *(CD_2Cl_2).*

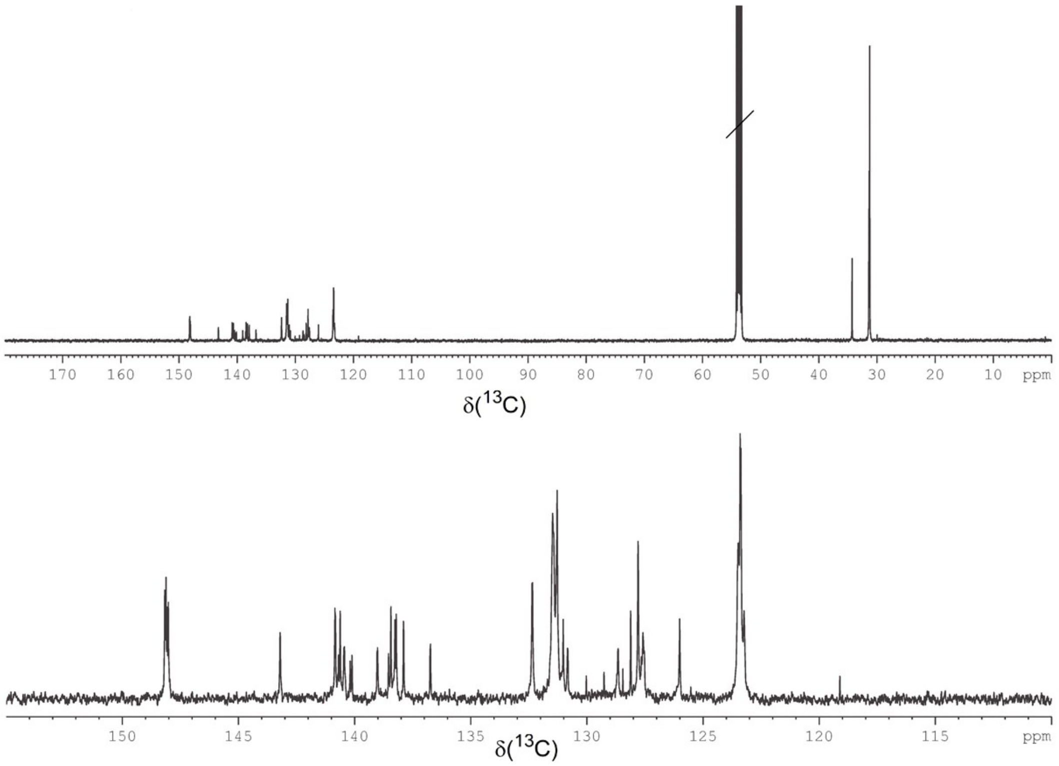

*Figure S15. ^{13}C NMR spectrum (125 MHz, CD_2Cl_2) of **6** (overview and enlarged region).*

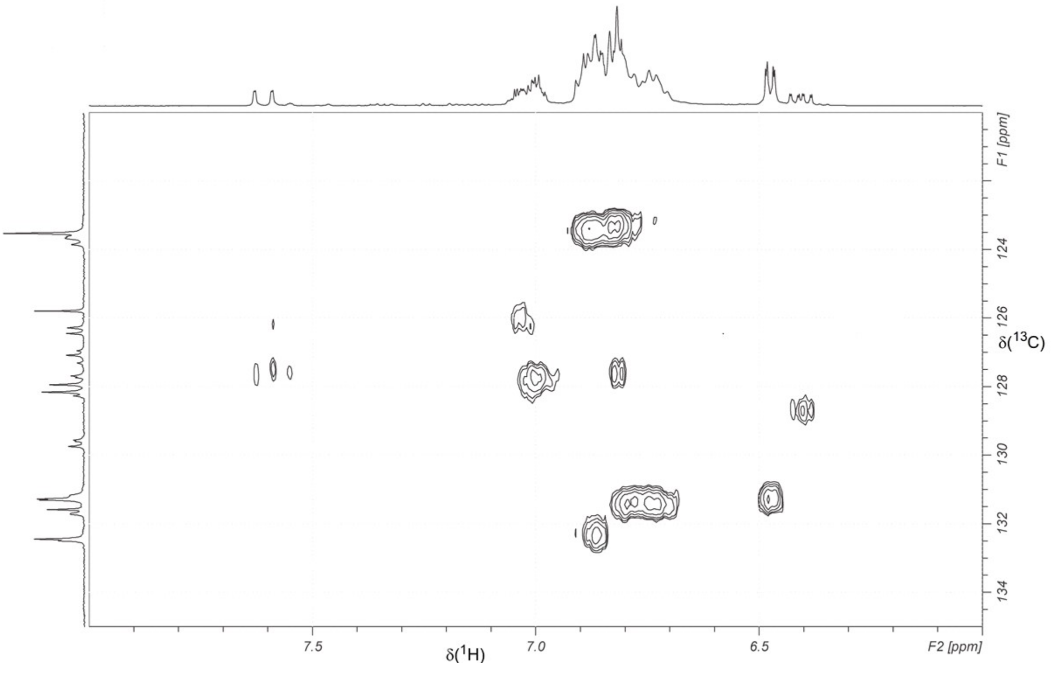

*Figure S16. HSQC spectrum (CD_2Cl_2) of **6** (region of aromatic carbons). The F1 trace is the DEPT135 spectrum.*

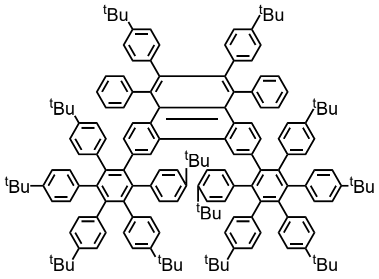

8

^{1}H (500 MHz, CD_2Cl_2): 7.58 (d, 1.7 Hz, 2H), 7.2-6.5 (overlapping signals), 6.48 (d, 8.3 Hz, 4H), 6.40 ppm (dd, 8.6 Hz, 1.7 Hz, 2H).

^{13}C (125 MHz, CD_2Cl_2): 148.2, 148.1, 148.0, 143.2, 140.9, 140.7, 140.6, 140.5, 140.1, 139.0, 138.5, 138.4, 138.2, 137.9, 136.9, 136.7, 132.3, 131.5, 131.3, 131.0, 129.8, 128.7, 128.1, 127.8, 127.6, 127.5, 126.2, 123.5, 123.4, 123.3, 34.3 (C_{tBu}), 31.4, 31.3 ppm (both CH_3).

Compound **8** is a mixture of several isomers formed by rotation around single bonds. The EXSY spectrum shows that exchange processes (rotation around single bonds) take place and contribute to the complexity of the spectra (Figure S19). The HSQC spectrum allows the identification of the aromatic CH carbons (Figure S21).

HR-MS MALDI (m/z): calculated for $C_{162}H_{172}$ $[M]^+$ 2118.3493; found, 2118.3459.

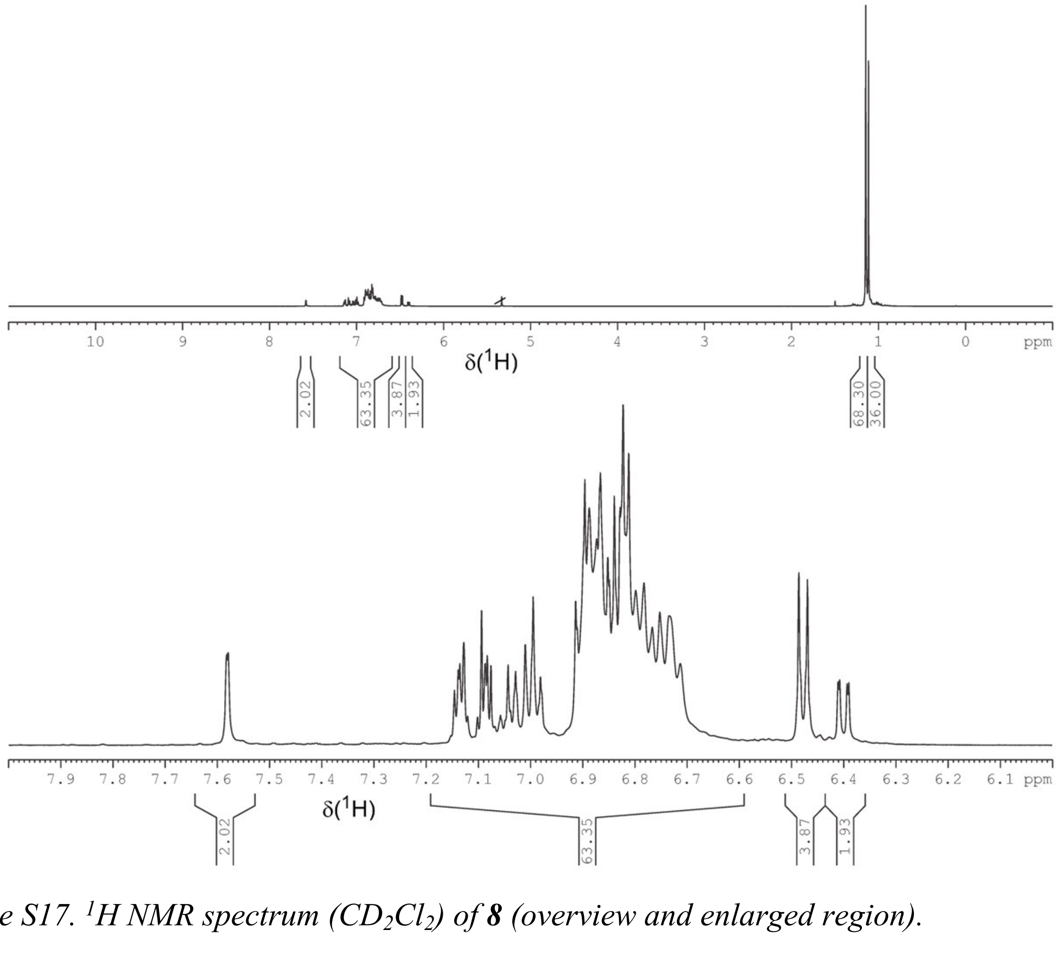

*Figure S17. ^{1}H NMR spectrum (CD_2Cl_2) of **8** (overview and enlarged region).*

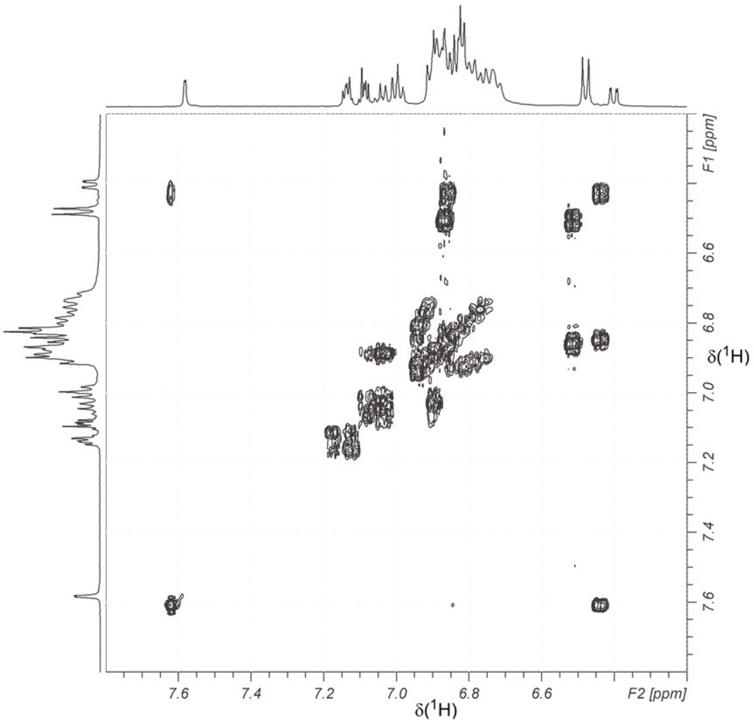

*Figure S18. COSY spectrum of **8** (CD_2Cl_2).*

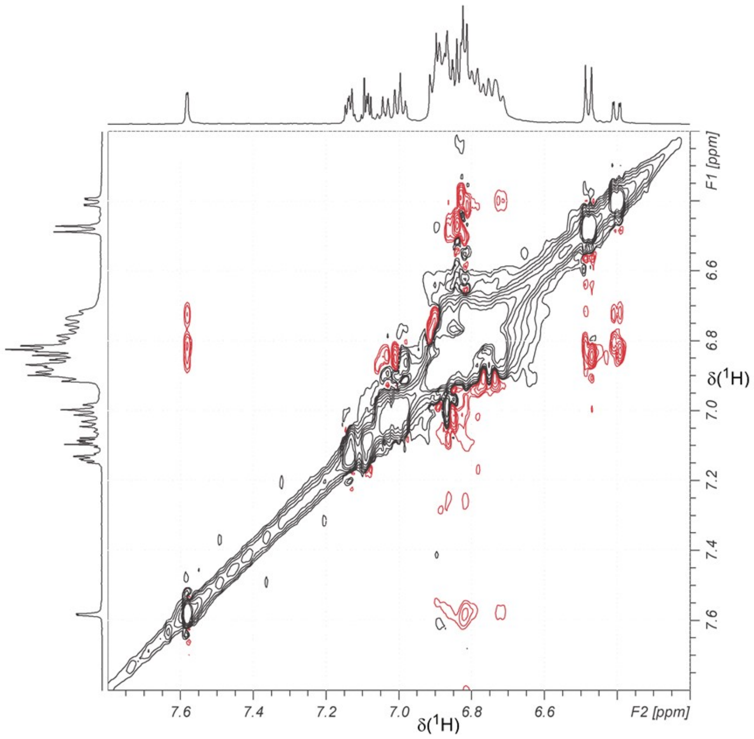

*Figure S19. EXSY (black correlations)/ROESY (red correlations) spectrum of **8** (CD_2Cl_2).*

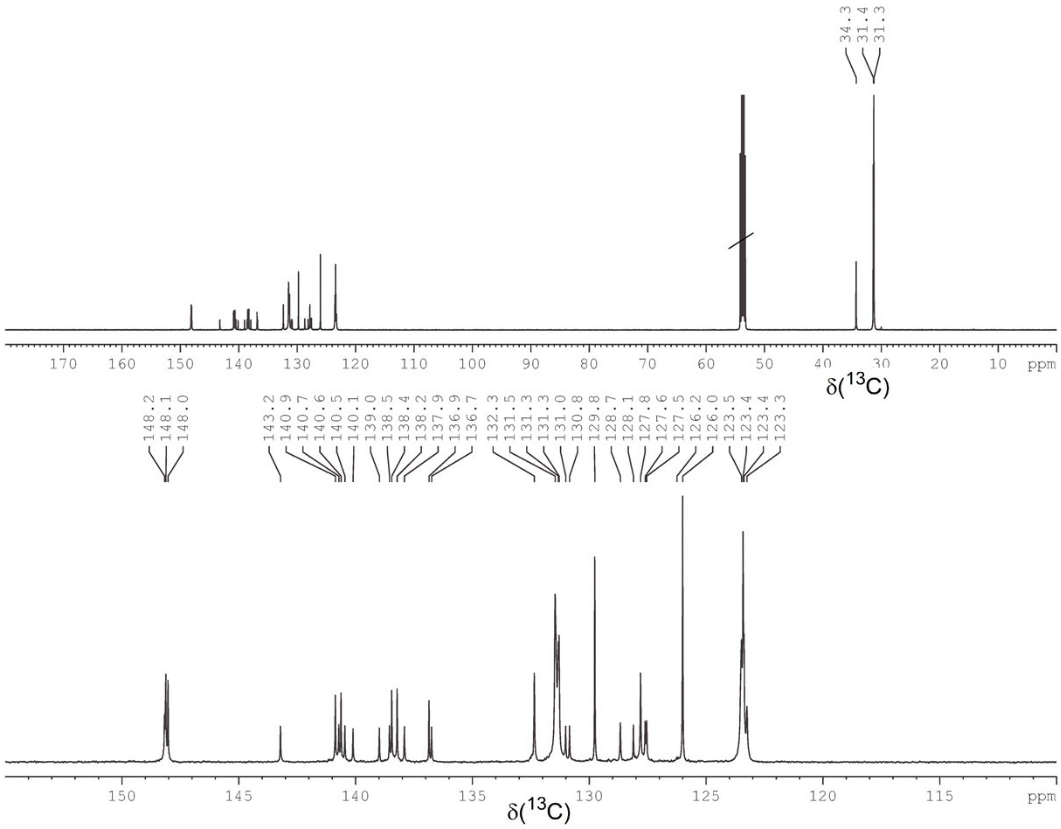

*Figure S20. ^{13}C NMR spectrum (CD_2Cl_2) of **8** (overview and enlarged region).*

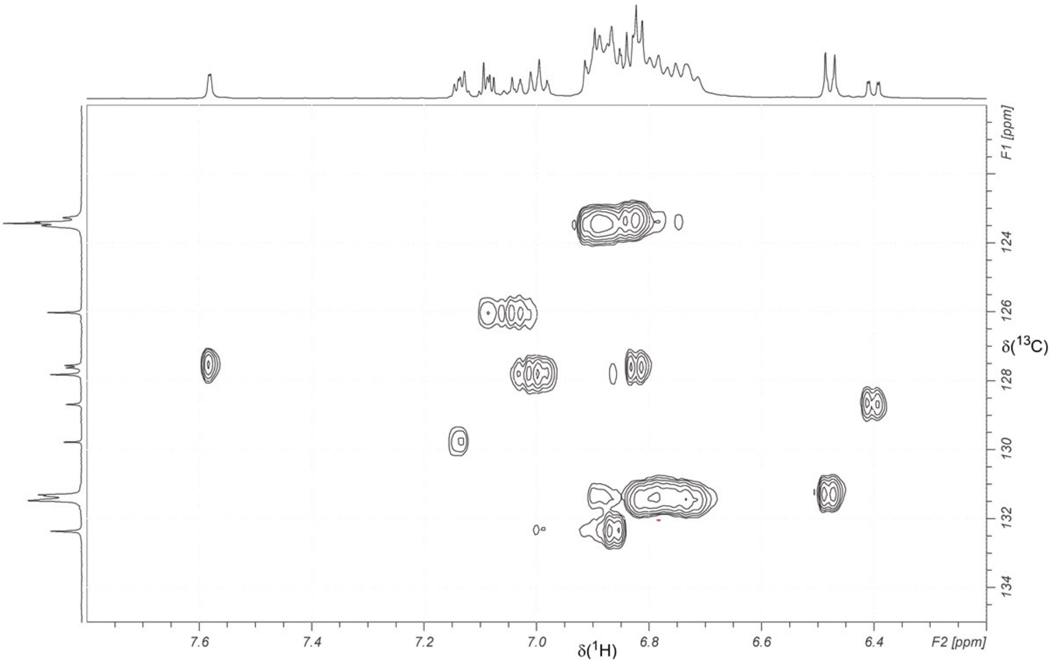

*Figure S21. HSQC spectrum (CD_2Cl_2) of **8** (region of aromatic carbons). The F1 trace is the DEPT135 spectrum.*

Synthesis of lateral extended double undecabenzo[7]helicene 1

In a 25 mL Schlenk flask, **6** (40 mg, 12 µmol) and DDQ (104 mg, 0.46 mmol) were dissolved in dry DCM (10 mL) under Ar atmosphere. The solution was cooled down to 0 °C with an ice-salt bath and stirred vigorously. After 10 min, 1 mL of CF_3SO_3H was injected through a syringe and the solution was stirred for 20 min. The reaction was quenched with excess of Et_3N and the solvent was evaporated in vacuo. The obtained residue was purified by prepared TLC with iso-hexane/DCM/CS_2 (10:2:1.7) as an eluent to afford **1** (16 mg, 41%) as a dark red solid.

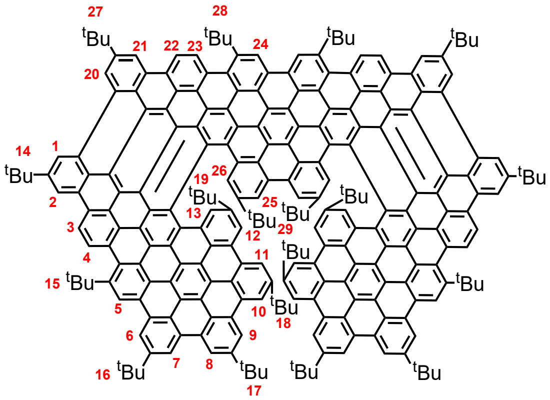

^{1}H NMR (500 MHz, $C_2D_2Cl_4$) δ 10.84 (s, 2H; 24), 10.28 (d, 9.3 Hz, 2H; 23), 10.27 (s, 2H; 5), 10.20 (d, 9.3 Hz, 2H; 22), 10.10 (s, 2H; 21), 10.28 (d, 9.3 Hz, 2H; 3), 10.02 (s, 2H; 2), 10.01 (d, 9.3 Hz, 2H; 4), 9.88 (s, 2H; 20), 9.86 (s, 2H; 1), 9.75 (s, 2H; 6), 9.49 (s, 2H; 7), 9.36 (s, 2H; 8), 9.11 (s, 2H; 9), 8.90 (s, 2H; 10), 8.60 (s, 2H; 26), 8.41 (s, 2H; 13), 8.22 (s, 2H; 11), 7.84 (s, 2H; 12), 7.55 (s, 2H; 25), 2.45 (s, 18H; 28), 2.24 (s, 18H; 15), 2.13 (s, 18H; 27), 2.10 (s, 18H; 14), 1.98 (s, 18H; 16), 1.71 (s, 18H; 17), 1.10 (s, 18H; 18), -0.35 (s, 18H; 19), -0.36 ppm (s, 18H; 29). The small $^4J_{HH}$ couplings are not resolved.

^{13}C NMR (125 MHz, $C_2D_2Cl_4$) data of CH and CH_3 carbons determined from the HSQC spectrum: δ 133.1 (4, 23), 126.9 (24), 126.2 (5), 124.5 (13), 123.3 (26), 121.1 (25), 120.8 (2), 120.7 (12, 21), 120.6 (20), 120.5 (1), 120.4 (6), 119.9 (8), 119.7 (7, 22), 119.6 (3, 9), 119.4 (11), 119.2 (10), 36.8 (28), 36.5 (15), 33.2 (14, 27), 33.0 (16), 32.7 (17), 32.2 (18), 30.5 (19), 30.4 ppm (29). The ^{13}C NMR spectrum is characterized by a large number of overlapping signals and it is less meaningful to list signals. For this reason, both the overview spectrum and the region of aromatic carbons are shown for reference (Figure S26).

HR-MS MALDI (m/z): calculated for $C_{258}H_{204}$ $[M]^+$ 3301.5874; found, 3301.5679.

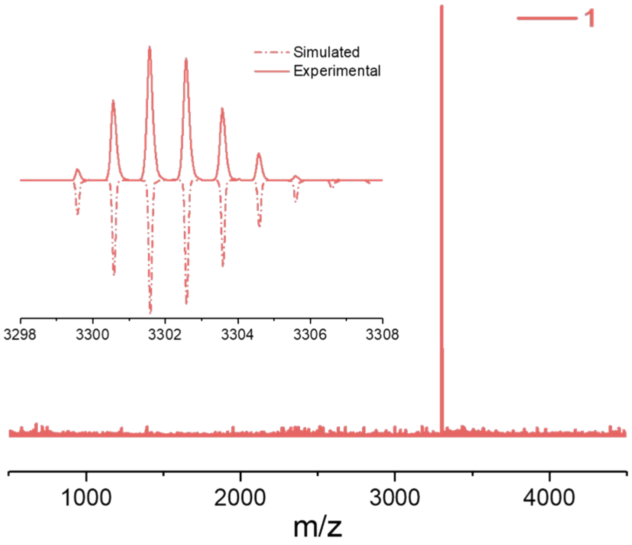

*Figure S22. HR-MALDI-TOF mass spectra of **1**.*

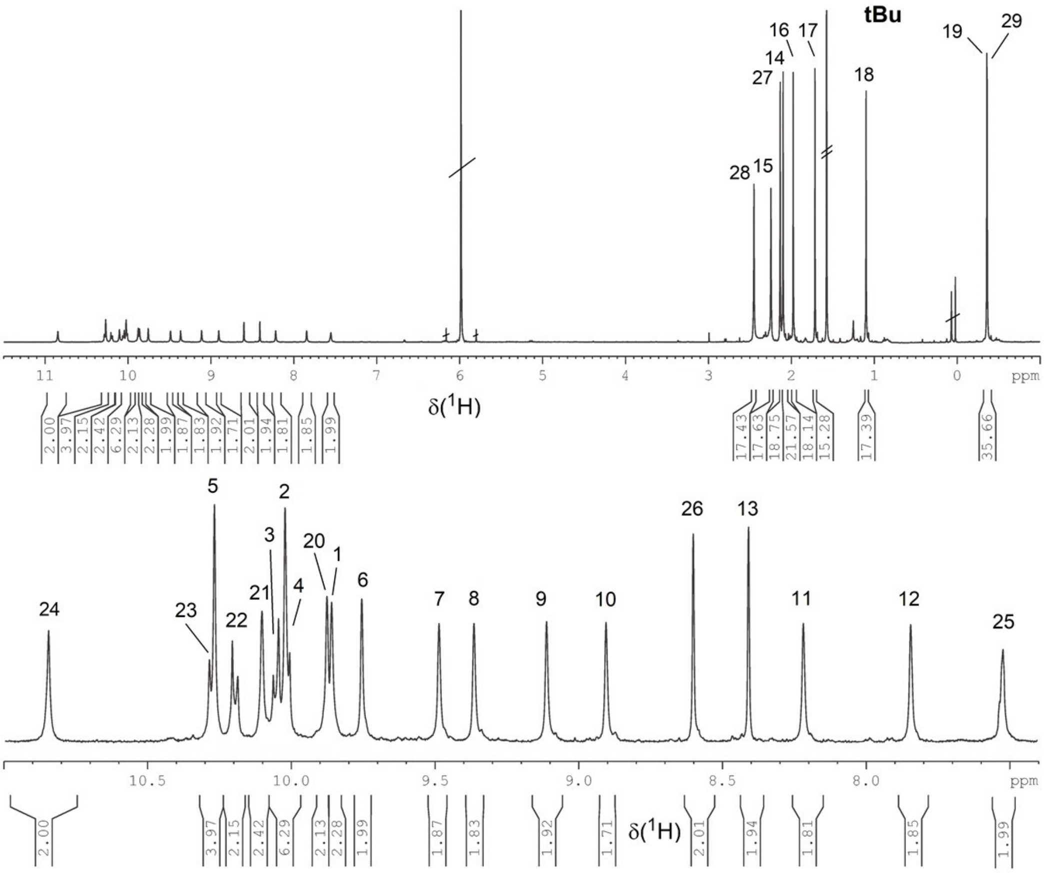

*Figure S23. ^{1}H NMR spectrum ($C_2D_2Cl_4$) of **1** (overview and enlarged region).*

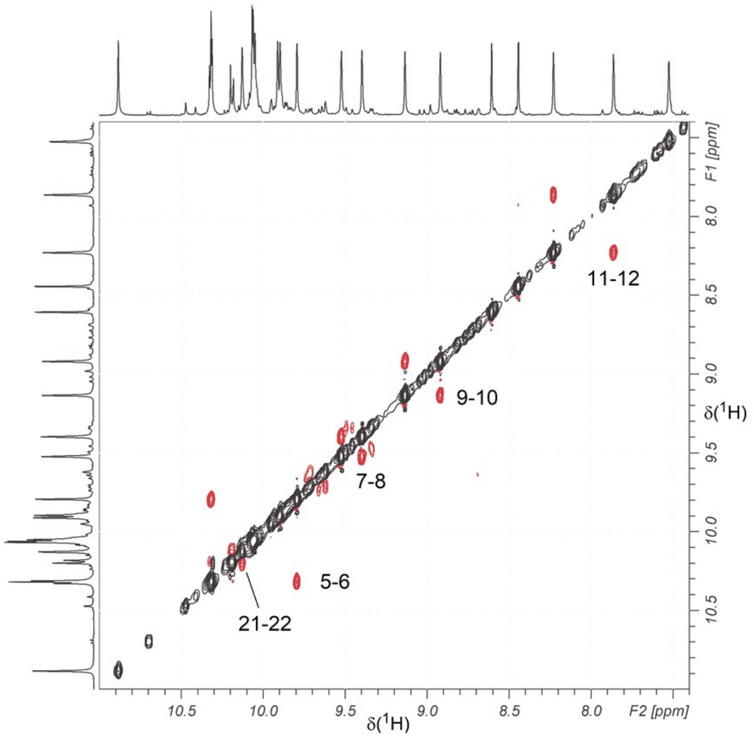

*Figure S24. ROESY (region of aromatic protons) spectrum of **1** ($C_2D_2Cl_4$).*

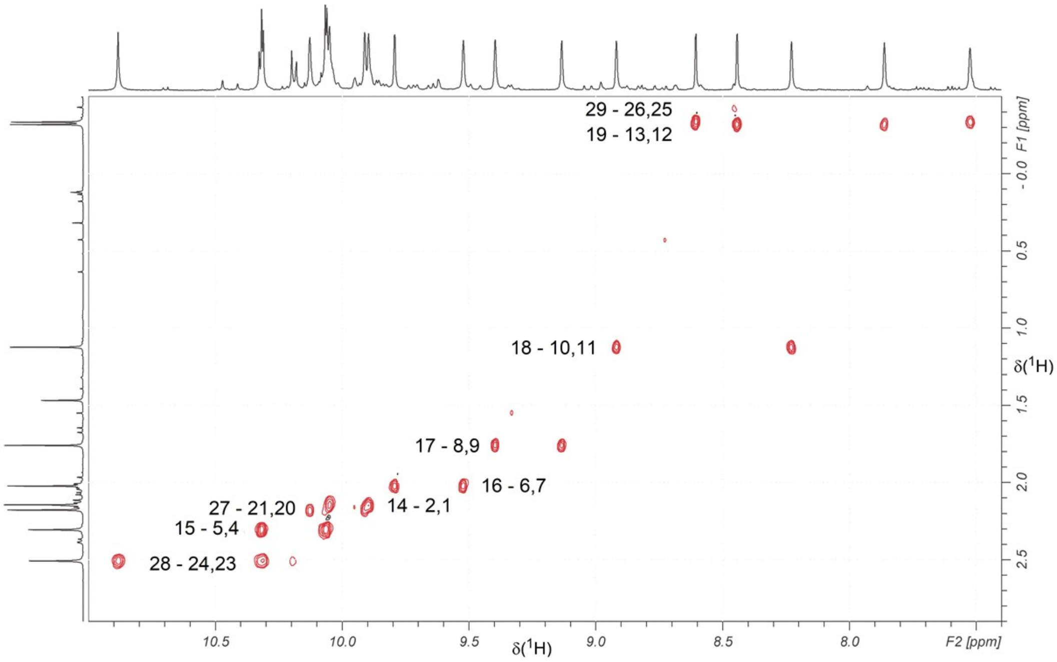

*Figure S25. ROESY spectrum (correlations between tBu groups and aromatic protons) of **1** ($C_2D_2Cl_4$).*

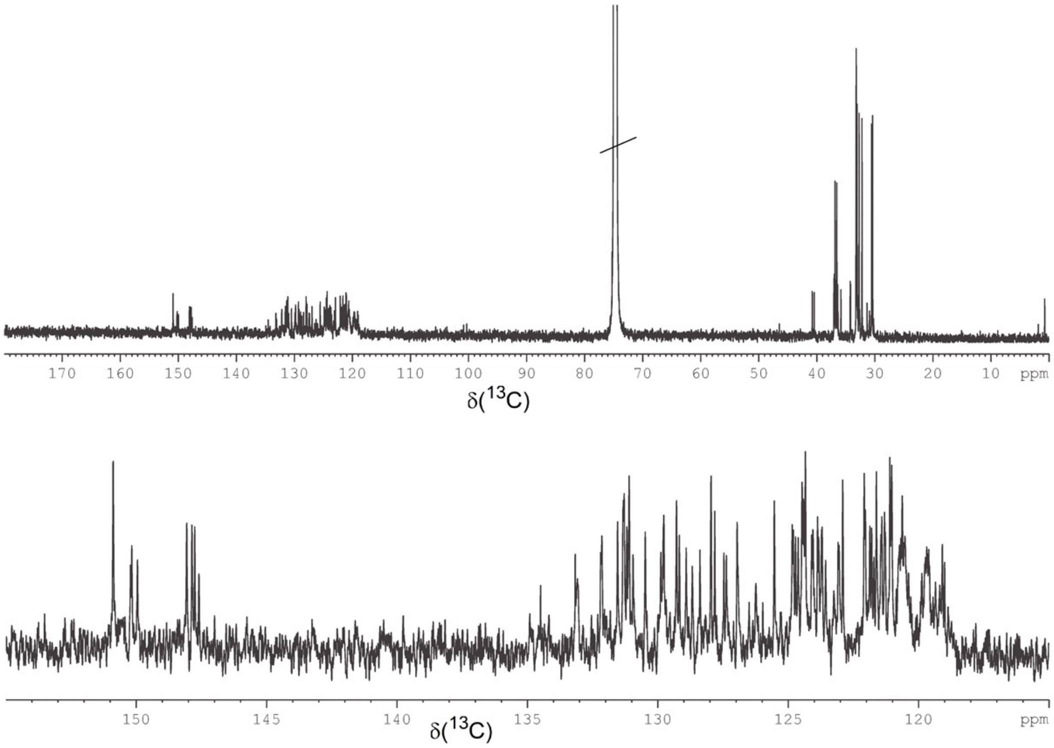

*Figure S26. ^{13}C NMR spectrum ($C_2D_2Cl_4$) of **1** (overview and enlarged region).*

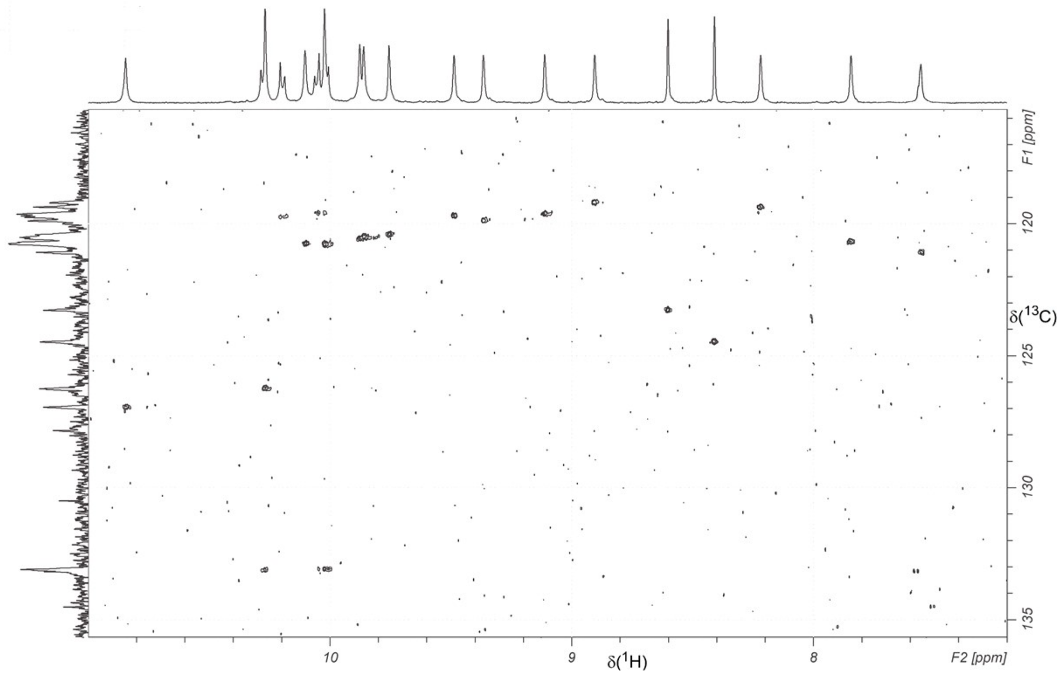

*Figure S27. HSQC spectrum ($C_2D_2Cl_2$) of **1** (region of aromatic carbons). The F1 trace is the DEPT135 spectrum.*

Synthesis of lateral extended undecabenzo[7]helicene 2

In a 25 mL Schlenk flask, **8** (25 mg, 12 µmol) and DDQ (61 mg, 0.27 mmol) were dissolved in dry DCM (10 mL) under Ar atmosphere. The solution was cooled down to 0 °C with an ice-salt bath and stirred vigorously. After 10 min, 0.5 mL of CF_3SO_3H was injected through a syringe and the solution was stirred for 20 min. The reaction was quenched with excess of Et_3N and the solvent was evaporated in vacuo. The obtained residue was purified by prepared TLC with iso-hexane/DCM/CS_2 (10:2:1) as an eluent to afford **2** (18 mg, 73%) as a dark red solid.

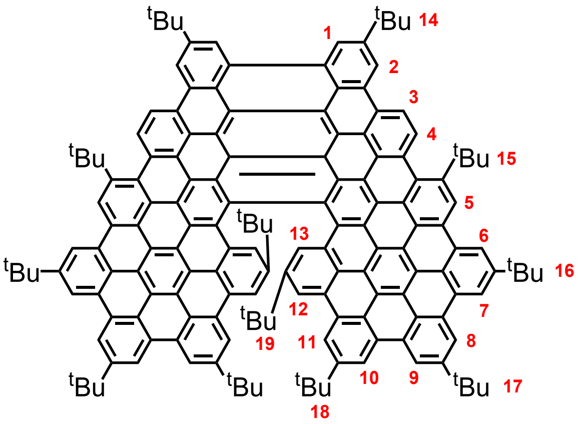

2

^{1}H NMR (500 MHz, $C_2D_2Cl_4$) δ 10.32 (s, 2H; 5), 10.04 (d, AB spin system, 2H; 3), 10.01 (d, AB spin system, 2H; 4), 9.98 (s, 2H; 2), 9.83 (s, 2H; 6), 9.81 (s, 2H; 1), 9.62 (s, 2H; 7), 9.54 (s, 2H; 8), 9.39 (s, 2H; 9), 9.26 (s, 2H; 10), 8.70 (s, 2H; 11), 8.59 (s, 2H; 13), 8.24 (s, 2H; 12), 2.25 (s, 18H; 15), 2.07 (s, 18H; 14), 2.05 (s, 18H; 16), 1.90 (s, 18H; 17), 1.60 (s, 18H; 18), -0.12 ppm (s, 18H; 19). The small $^{4}J_{HH}$ couplings are not resolved.

^{13}C NMR (125 MHz, $C_2D_2Cl_4$) data of CH and CH_3 carbons determined from the HSQC spectrum: δ 133.0 (4), 126.2 (5), 126.2 (5), 124.7 (12), 120.9 (13), 120.7 (2), 120.5 (1, 6), 120.1 (8), 120.0 (9), 119.9 (11), 119.8 (7), 119.6 (3), 119.4 (10), 36.5 (15), 33.1 (14, 16), 32.9 (17), 32.7 (18), 30.6 ppm (19). The ^{13}C NMR spectrum is characterized by a large number of overlapping signals and it is less meaningful to list signals. For this reason, both the overview spectrum and the region of aromatic carbons are shown for reference (Figure S32).

HR-MS MALDI (m/z): calculated for $C_{162}H_{134}$ $[M]^{+}$ 2080.0519; found, 2080.0510.

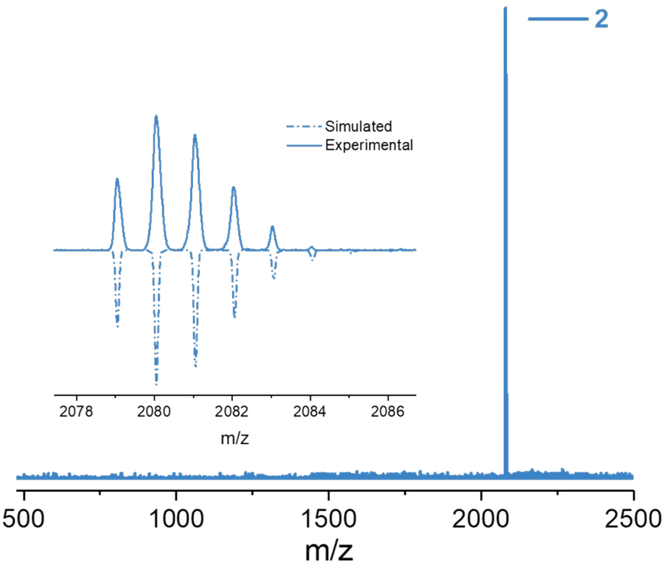

*Figure S28. HR-MALDI-TOF mass spectra of **2**.*

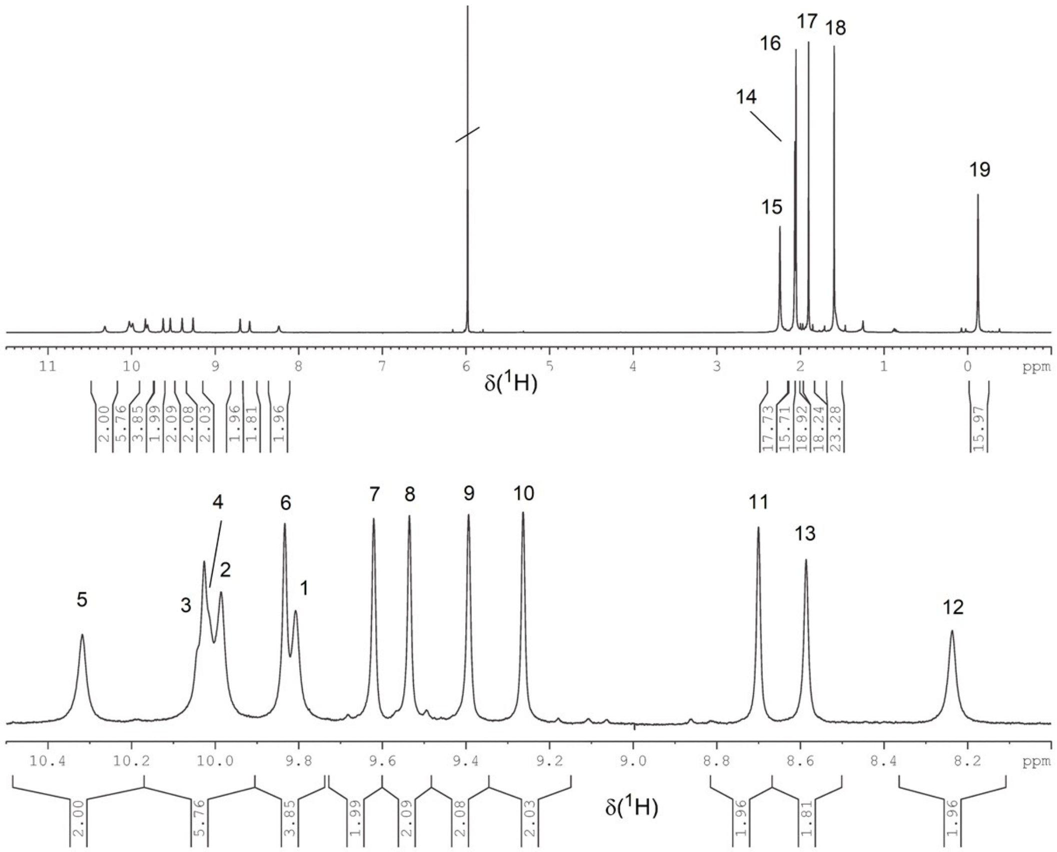

*Figure S29. ¹H NMR spectrum ($C_2D_2Cl_4$) of **2** (overview and enlarged region).*

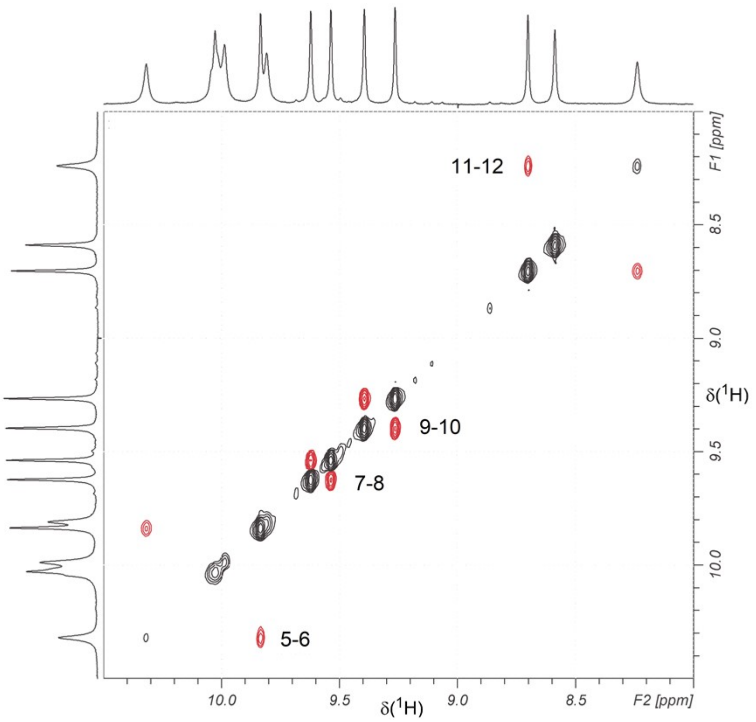

*Figure S30. ROESY (region of aromatic protons) spectrum of **2** ($C_2D_2Cl_4$).*

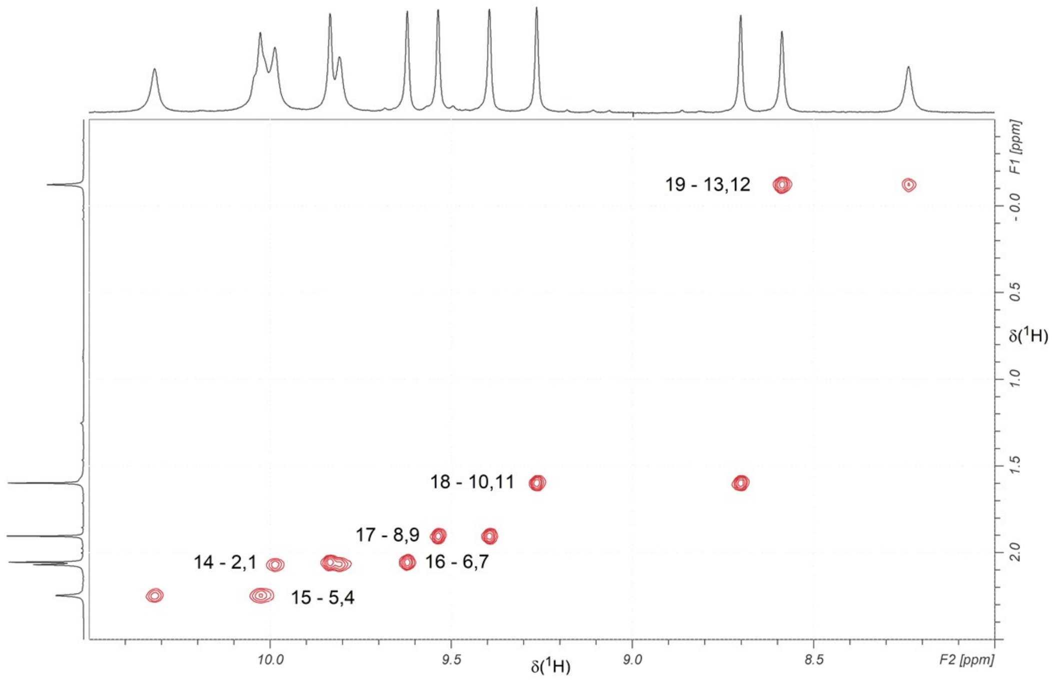

*Figure S31. ROESY spectrum (correlations between tBu groups and aromatic protons) of **2** ($C_2D_2Cl_4$).*

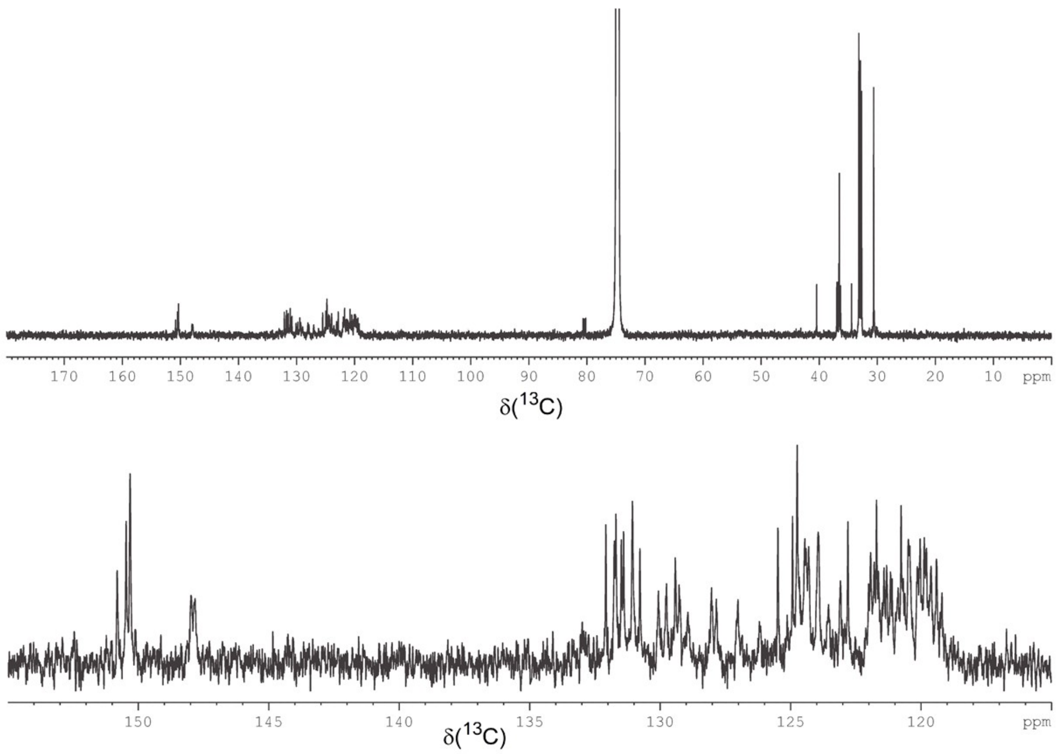

Figure S32. [13]*C NMR spectrum (*$C_2D_2Cl_4$*) of* ***2*** *(overview and enlarged region).*

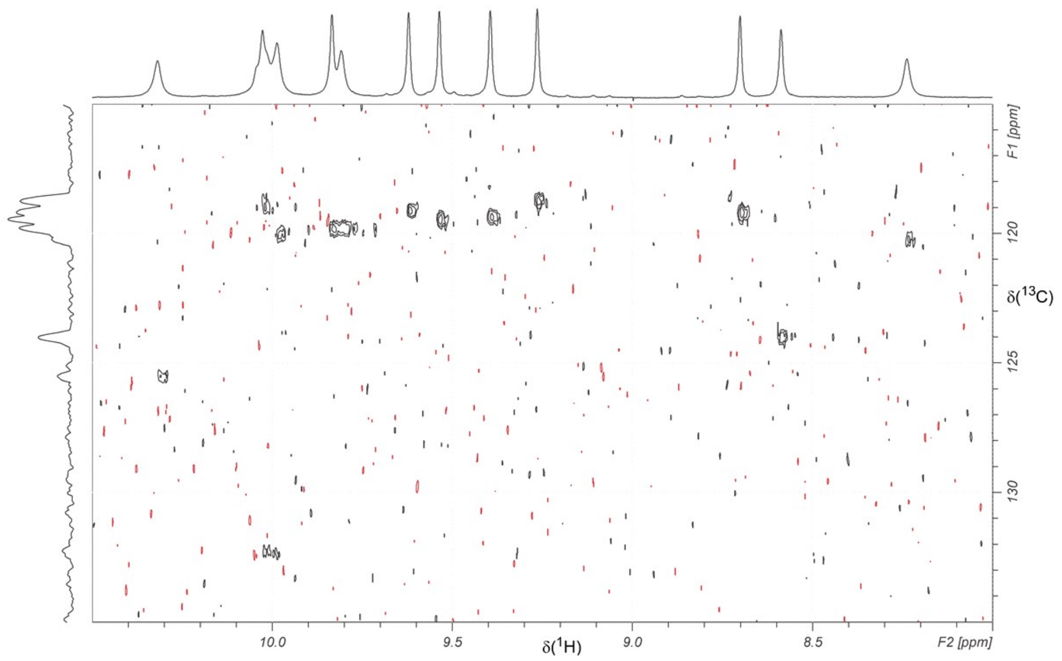

Figure S33. HSQC spectrum ($C_2D_2Cl_2$*) of* ***2*** *(region of aromatic carbons).*

X-ray crystallographic analysis of 1

Single crystal of compound **1** was obtained by slow diffusion of methanol into the chlorobenzene solution. X-ray diffraction data collection was carried out at the BESSY storage ring (BL14.2, Berlin-Adlershof, Germany).[3] XDSAPP2.0 suite was employed for data processing.[4-5] The structure was solved by direct methods and refined by SHELXL-2019.[6] Hydrogen atoms were added geometrically and refined with a riding model.

Table S1. Crystallographic data and details of the structure refinements of NG **1**.

**Crystal**	**1**
**Moiety formula**	$C_{258}H_{202}$
**Formula weight**	3302.18
**Crystal size, mm**	0.01x0.01x0.05
**Crystal system**	triclinic
**Space group**	$P\bar{1}$
***a*, Å**	17.520(4)
***b*, Å**	19.890(4)
***c*, Å**	34.140(7)
***α*, deg**	103.49(3)
***β*, deg**	94.55(3)
***γ*, deg**	112.42(3)
**Volume, Å^3**	10505(4)
Z	2
$D_{calcd.}$, g cm^{-3}	1.044
F_{000}	3500
***T*, K**	100
**Radiation (λ, Å)**	Synchrotron (0.7749)
**μ, mm^{-1}**	0.071
**2Θ range (°)**	2.498 to 51.022
**Index ranges**	$-19 \leq h \leq 19$, $-22 \leq k \leq 22$, $-37 \leq l \leq 37$
**no. of collected reflections**	97176
**no. of unique ref. (R_{int})**	26934 (0.1171)
**Data/restraints/parameters**	11013/0/2377
**R_1, wR_2 [obs I>2σ (I)]**	0.0908, 0.2354
**R_1, wR_2 (all data)**	0.1765, 0.2873
**residual peak/hole, e. Å^{-3}**	0.235/-0.202
**Goodness-of-fit on F^2**	0.936
**CCDC**	2412066

3. Optical properties and DFT calculations

Density functional theory (DFT) calculation was performed using the Gaussian 16 program.[7] The B3LYP functional with Grimme's D3 correction (Becke–Johnson damping)[8] was used for geometry optimization in the ground state. The 6-31G(d) basis set was used. All geometry optimization was done in the gas phase and based on the single crystal structures. In order to simulate the UV-Vis spectra of the molecules TD-DFT calculations were carried by using B3LYP functional and 6-31G(d) basis set. For better comparison to the experimental absorption spectra the polarity of the solvent dichloromethane was added.

Molar extinction coefficient of 1-*PP*/*MM*

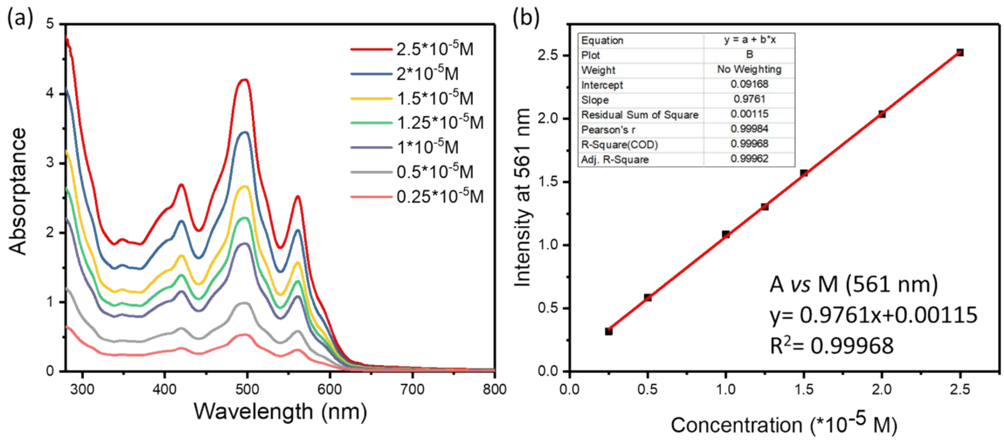

*Figure S34. (a) Absorbance spectra of **1**-PP/MM at different concentrations in anhydrous DCM; (b) Absorbance vs concentration plot of compound **1**-PP/MM at ~561nm.*

Molar extinction coefficient of *2-P/M*

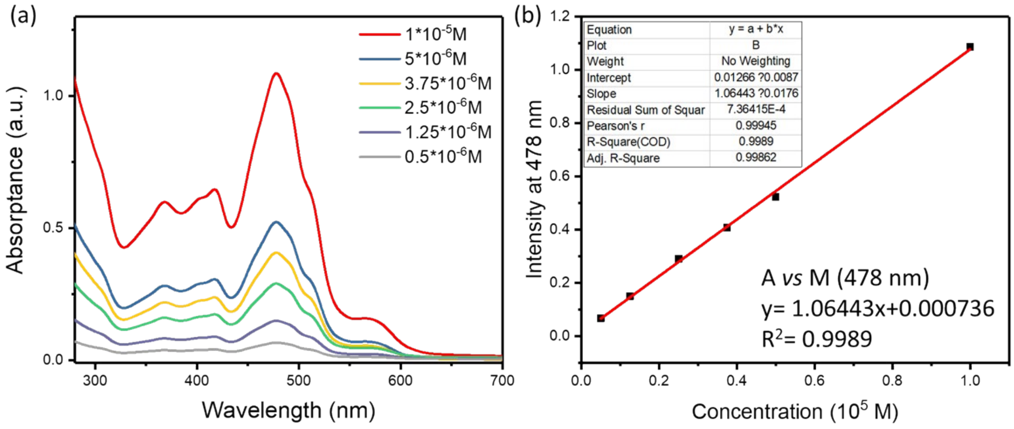

*Figure S35. (a) Absorbance spectra of **2**-P/M at different concentrations in anhydrous DCM; (b) Absorbance vs concentration plot of compound **2**-P/M at ~478 nm.*

HPLC curve of 1-*PP*/*MM*

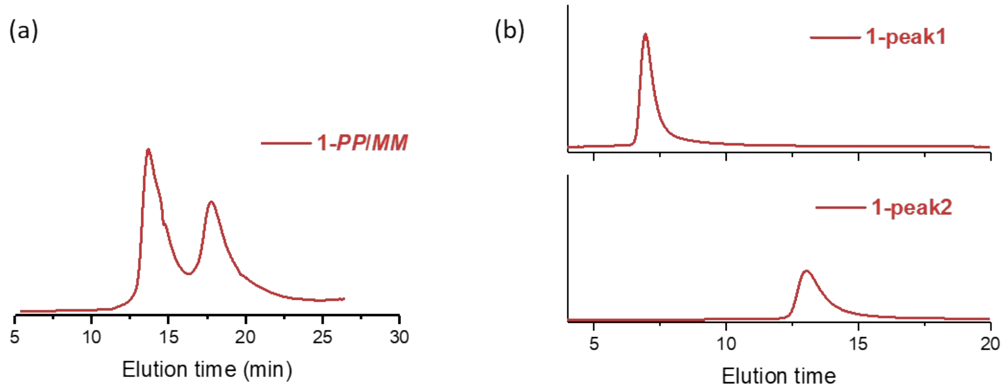

Figure S36. (a) Chiral separation of ***1****-PP/MM conducted on a Shimadzu FCV-20AH2 instrument with a (R,R)-Whelk-O 1 5 μm Kromasil column (250 mm × 21.1 mm). Eluent:* $CHCl_3$*/n-Hexane = 30/70 (v/v), 14 mL/min. The detection wavelength was 400 nm. (b) Analytical HPLC traces of two enantiomers after chirality separation with a Whelk-O 1 5 μm Kromasil column (250 mm × 4.6 mm).*

HPLC curve of 2-*P*/*M*

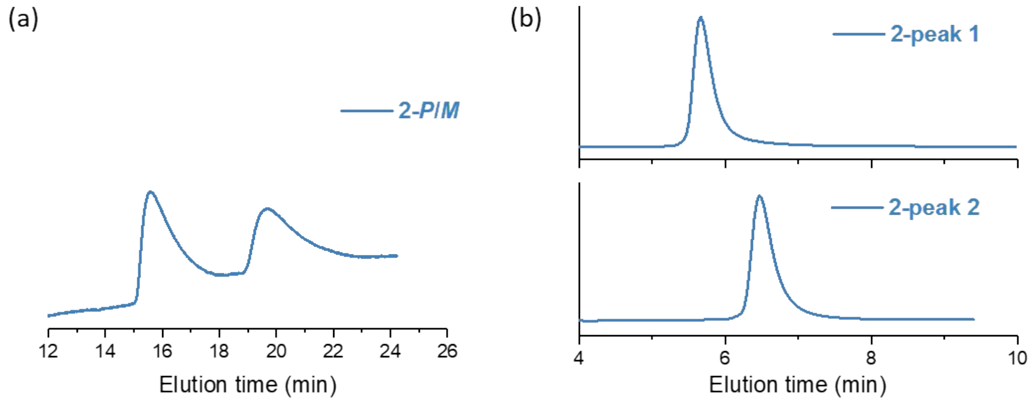

Figure S37. (a) Chiral separation of ***2****-P/M conducted on a Shimadzu FCV-20AH2 instrument with a (R,R)-Whelk-O 1 5 μm Kromasil column (250 mm × 21.1 mm). Eluent:* $CHCl_3$*/n-Hexane = 20/80 (v/v), 14 mL/min. The detection wavelength was 400 nm. (b) Analytical HPLC traces of two enantiomers after chirality separation with a Whelk-O 1 5 μm Kromasil column (250 mm × 4.6 mm).*

Molecular frontier orbitals

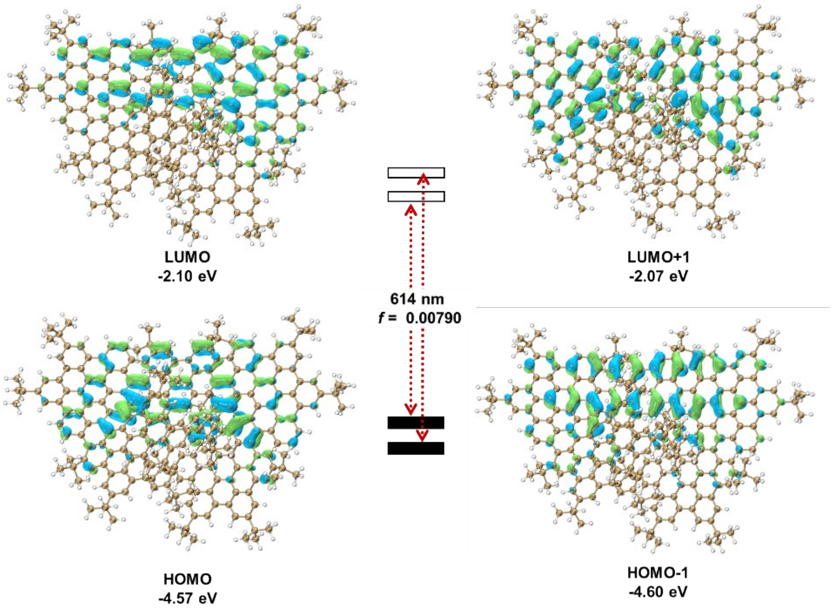

*Figure S38. Molecular frontier orbitals and the energy diagrams of **1** calculated at the B3LYP-D3(BJ)/6-31G(d) level.*

Spectrum calculation of 1-*PP*

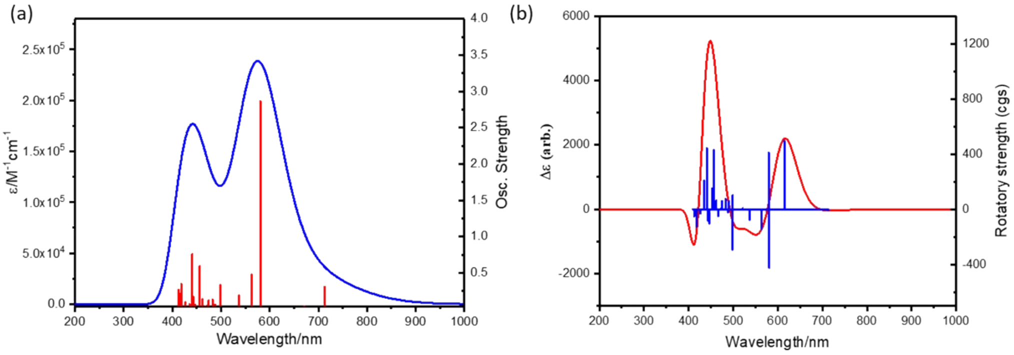

Figure S39. UV-vis and CD spectra calculation at the B3LYP-D3(BJ)/6-31G(d) level.

Transition dipole moments

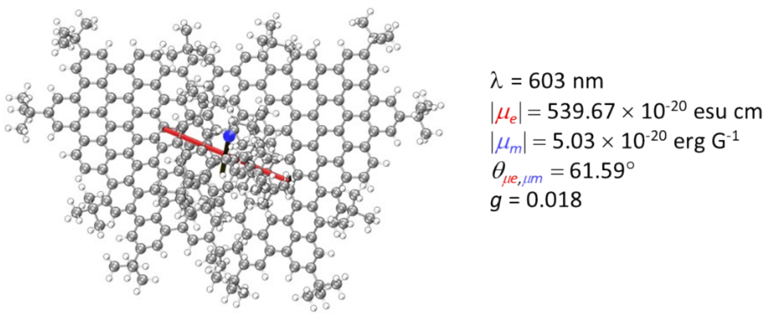

*Figure S40. Transition dipole moments of **1**-PP for the S0→S2 transition.*

4. mc-AFM measurements

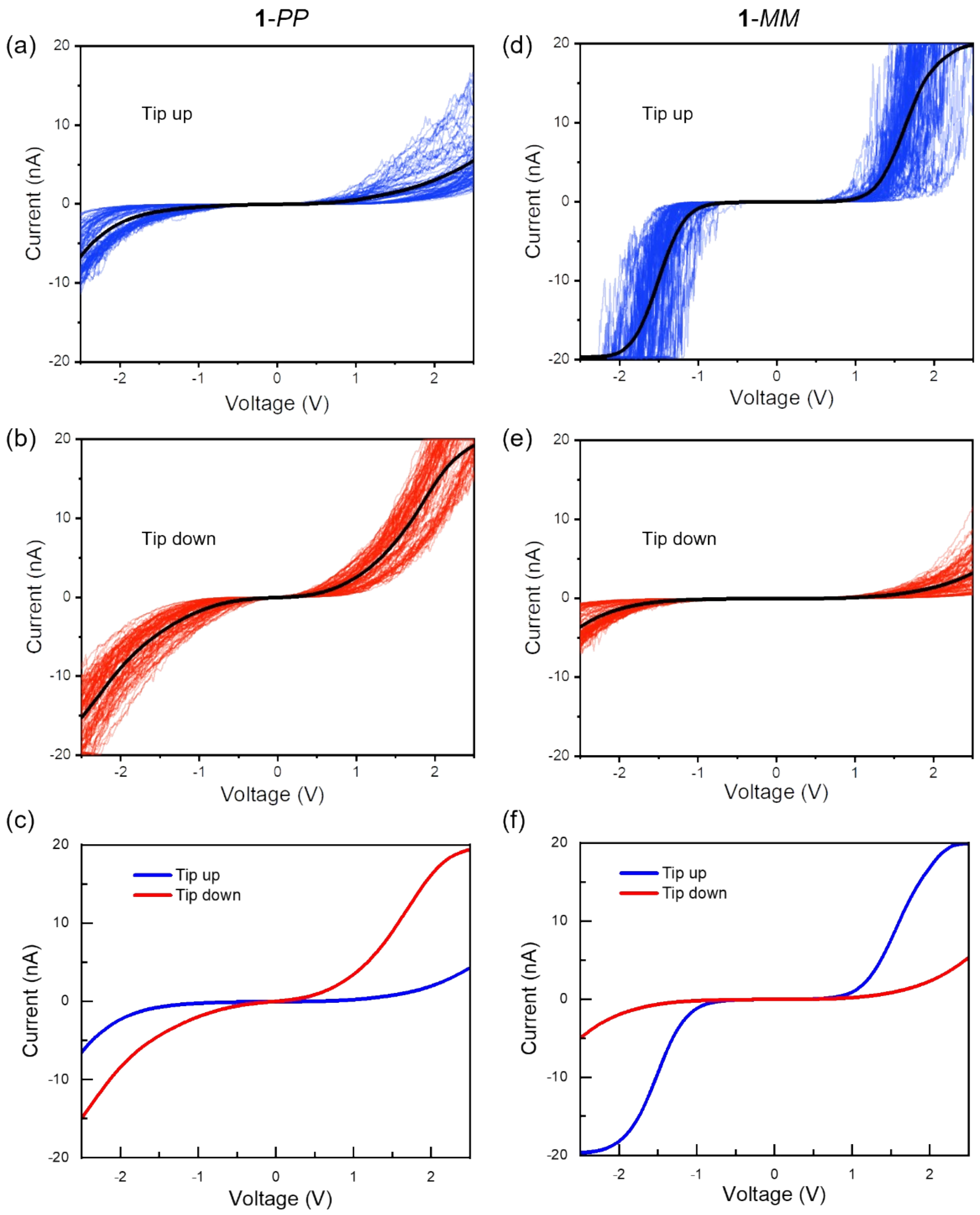

*Figure S41. Spin-dependent conduction through the **1**-PP (a-c) and **1**-MM (d-f) thin films. Measurements were performed by magnetic-conductive-probe AFM (mCP-AFM) with a ferromagnetic (Co-Cr-coated) tip. The tip can be magnetized by a permanent magnet with different magnetization directions (field-up or field-down with respect to the substrate), and the magnetized tip is subsequently used in the measurements.*

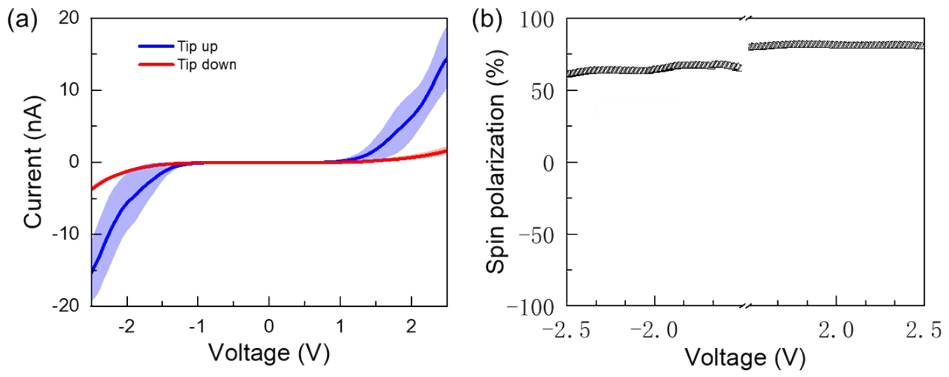

*Figure S42. Spin-dependent conduction through the **2**-M thin films. (a) The average current as a function of applied voltage for **2**-M when the tip is magnetized in the upward (blue line) and downward (red line) directions. (b) Spin polarization values of **2**-M as a function of applied bias voltage.*

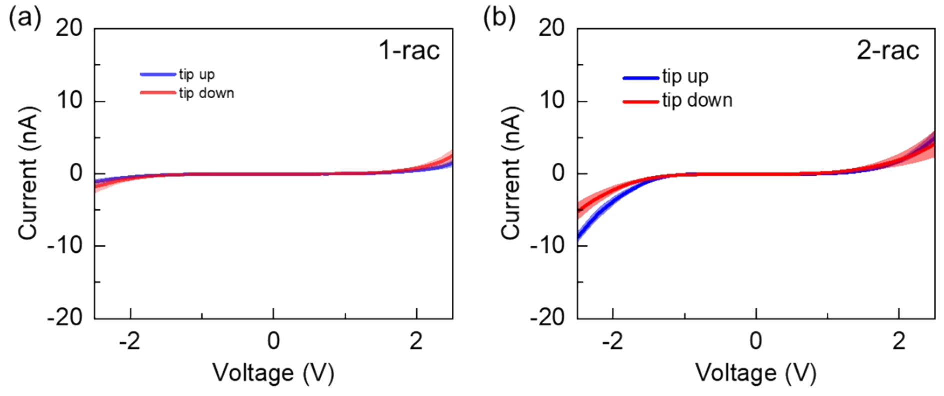

*Figure S43. mc-AFM measurements of racemic mixture of NG **1**-PP/MM (a) and NG **2**-P/M (b).*

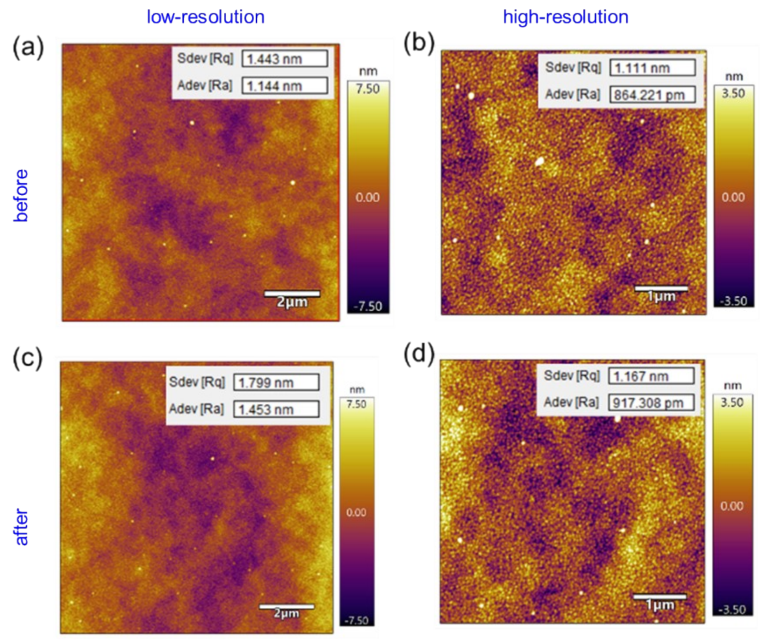

Figure S44. AFM images of Au surface (a, b) before and (c, d) after spin coating of chiral molecules with concentration of 10^{-6} M in DCM.

5. Magnetoresistance measurements

Device fabrication

The spin-valve-like devices consist of 3 key components: bottom electrodes, NG molecular layer and top electrodes. There are 5 main steps to fabricate devices. (1) The bottom electrodes are made of $Co_{20}Fe_{60}B_{20}$ (5 nm)\MgO (2.5 nm) thin films deposited by magnetron sputtering on a Si substrate with thermal oxide layer and a non-magnetic buffer layer with a base pressure of 1×10^{-9} Torr. (2) After deposition, the extended films are fabricated into bottom electrode stripes with standard ultraviolet lithography (UVL) by Heidelberg Instruments MLA 150. Ion beam etching and aluminum oxide (AlO_x) deposition is performed in Scia coat 200. The AlO_x helps isolate the bottom electrodes from the top electrodes. Then, the photoresist is removed by acetone in ultrasonic cleaner for 5 min and ozone cleaner for 3 min. (3) Just after the cleaning, the samples are put into the **1**-*PP* or **1**-*MM* solution in tetrahydrofuran (THF) and kept for 48 hours. The samples are taken out and mounted on a spin-coater. A one-minute spin-coating with 1000 round per minute is used to uniform the NG layer. (4) A 25-nm platinum layer is deposited on the whole sample. (5) The UVL process is used to fabricate the platinum layer into top electrode stripes. The resist is mechanically removed when wire-bonding to avoid violation of the NG layer by organic solutions.

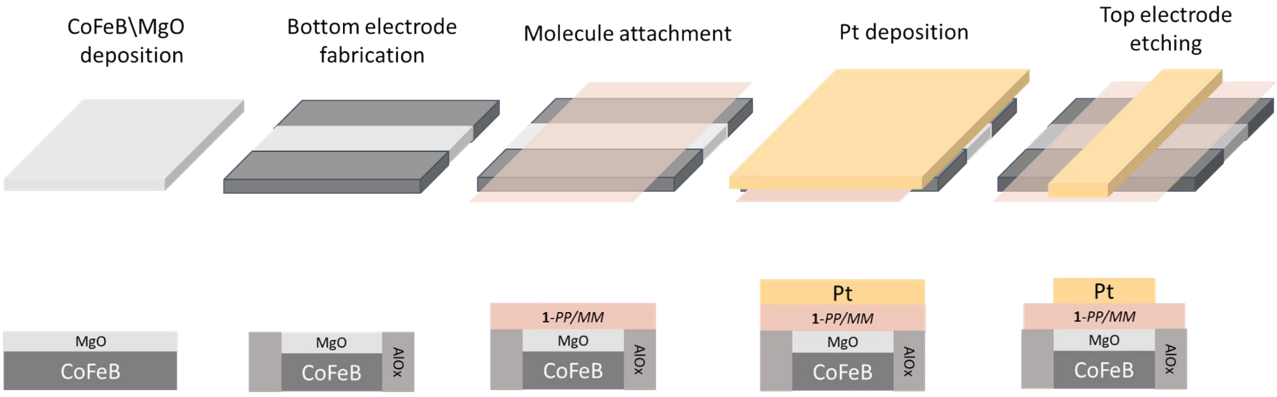

Figure S45. Device fabrication process. The spin-valve-like devices are made by 5 steps following the sequence from left to right.

Electrical measurement

The device is sticked to the sample holder and connected by Al wire. The sample holder is mounted in Quantum Design PPMS-9T, which offers the temperature and magnetic field conditions. The dc current is loaded by Keithley 6221 and the response voltage is picked up by Keithley 2182A.

6. Computational methodology and spin transport calculations

Computational Methodology

Electronic structure calculations. We have used the charge-selfconsistent Density-Functional Tight-Binding (DFTB+) methodology[9] with the *auorg* set of Slater-Koster parameters to perform the structural optimization of the NG **1** helical molecule on a Au(111) terminated surface. For testing purposes, two different van-der-Waals corrections, Grimme (D3) [10] and Tkatchenko-Scheffler (TS)[11], as implemented in the DFTB+ code, were used. Periodic boundary conditions in the x-y plane were imposed and a vacuum gap of 5 nm in the z-direction to avoid interactions between structures in neighboring supercells along the z-axis. A supercell contained 1060 atoms and we included valence 6s-, 6p-, and 5d-states for Gold and valence 2s- and 2p-states for Carbon atoms. Convergence was achieved when the total force acting on the atoms was below a cut-off of 10^{-5} a.u.

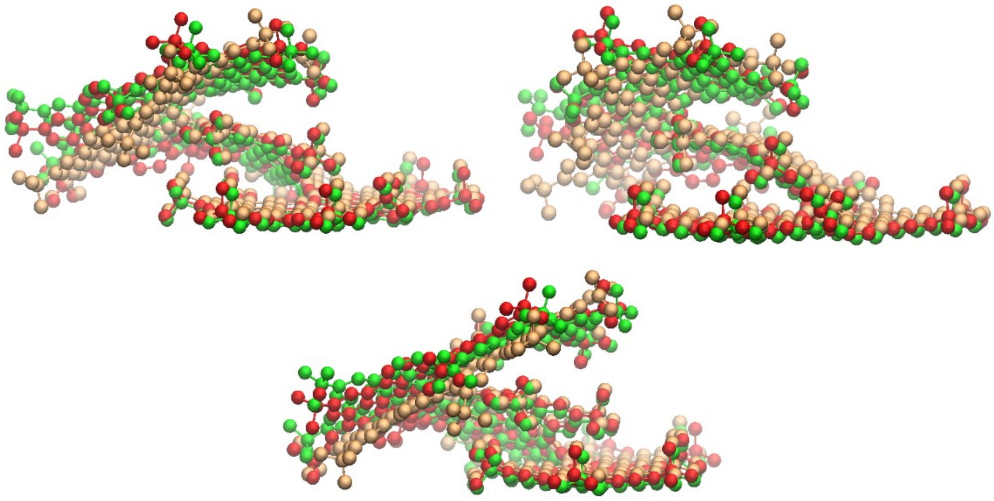

Figure S46. Superposition of the three relaxed configurations investigated in the manuscript. The xyz coordinates were extracted after relaxing each of them on the Au(111) substrate. The three geometries were then centered at their corresponding center of mass to make the visualization easier. For the same reason the tert-butyl groups were removed and only carbon atoms were left. The basal plane in direct contact with the substrate is always shown at the bottom of each figure. The different colors only serve to enhance the contrast. The three panels show different perspectives to better highlight structural differences.

Spin-dependent transport calculations

We have computed the spin polarization using the Landauer theory expressed in terms of Green functions, see e.g. [12]. Since we are not considering spin-orbit interactions, the generic form of the Hamiltonian matrix for the molecule in spin space will look like

$$H = \begin{pmatrix} H_u & 0 \\ 0 & H_d \end{pmatrix}, \qquad (1)$$

where $H_{u,d}$ are Hamiltonian block matrices for the spin-up (u) and -down (d) channels, respectively. Previous model calculations and general symmetry arguments[13-16] have shown that *even* in the presence of spin-orbit coupling, a non-zero spin polarization is only obtained if

time reversal symmetry (TRS) or reciprocity are broken (induced e.g. by decoherence processes). This means, in the presence of TRS $H_u = H_d$ and no spin polarization can be obtained. To account for the breaking of TRS taking place in transport experiments (resulting from the applied voltage and/or environmental decoherence) and of the CISS effect, we have adopted a phenomenological approach to the problem: our starting point is that, based on the results presented in the experimental measurements of the spin polarization of the electrical current (Fig. 2f in the main text), TRS is broken and the CISS effect is present in the system in question, i.e. we are not attempting to provide a microscopic picture of all the factors which might lead to it and which have been addressed in other publications, see e.g. the review in Ref. [17].

Hence, we assume that TRS breaking will translate into an asymmetry of the electronic coupling matrix elements for the spin-up and spin-down channels of the full molecular Hamiltonian matrix $\boldsymbol{H}$ in Eq. (1). This asymmetry will be accounted for with a phenomenological parameter β, which modifies the hopping matrix elements such that $H_{nm,u} = \beta H_{nm,d}$ for $n \neq m$, and β being only slightly different from 1. Notice that a similar approach was used in Ref.[16]. Based on this central approximation, the total molecular Hamiltonian is still block diagonal in spin space but with $\boldsymbol{H}_u \neq \boldsymbol{H}_d$.

Now, in a second step, we need to account for the CISS effect, i.e., different spins injected via a polarized electrode are transmitted in different ways across the chiral molecule. Since we compare two different setups to mimic the two experimental situations: one injecting spin-up electrons from the magnetic tip into the molecule, which we denote with (↑), and the other one, where spin-down electrons are similarly injected from the magnetic tip into the molecule. This second setup is symbolically denoted as (↓). Here, spin up and down refers to a spin quantization axis provided by the magnetized Ni tip.

As a result of these two different setups, two different β parameters will be required, which we denote as $\beta_{\uparrow,\downarrow}$. The fact that $\beta_\uparrow \neq \beta_\downarrow$ accounts now for the presence of the CISS effect, i.e., spin selectivity, since one of the spin components will be easier transferred in one of the two computational setups than in the other. With their help, we can define the asymmetry factor $\delta = |\beta_\uparrow - \beta_\downarrow|/(\beta_\uparrow + \beta_\downarrow)$ introduced in the main text.

The bare (i.e. non-scaled with β) Hamiltonian and Overlap matrix elements for the NG **1** molecule are extracted from single point calculations using the DFTB+ code. Hereby, we used the molecular conformation already relaxed on the Au(111) surface, so that the influence of the substrate on the molecular geometry is appropriately caught (the substrate details do not play a role within the wide-band approximation used here, see below). As a result, the total Landauer transmission can be decomposed into separate components $T_s(E)$, s=↑,↓, for the two transport setups, (↑) and (↓), see Eq. (3) below, and with it, an energy-dependent spin asymmetry factor $\Delta(E)$ can, thus, be defined as:

$$\Delta(E) = \frac{T_\uparrow(E) - T_\downarrow(E)}{T_\uparrow(E) + T_\downarrow(E)}, \qquad (2)$$

which is a measure for the spin polarization power of the molecule. Here, the transmission is defined as (T=top electrode, B=bottom electrode):

$$T_s(E) = Tr\{\Gamma^T(E)G_s(E)\Gamma^B(E)G_s^+(E)\}. \quad (3)$$

The (retarded) Green function $G_s^{-1}(E) = ES - H_s - \Sigma^T(E) - \Sigma^B(E)$. The complex self-energies $\Sigma^T(E)$ and $\Sigma^B(E)$ encode the electronic structure of the T (magnetic tip) and B (Au substrate) electrodes as well as structural and electronic features of the molecule-electrode interface. The spectral matrices $\Gamma^{T,B}(E) = i(\Sigma^{T,B}(E) - (\Sigma^{T,B}(E))^+)$ are in general energy-dependent, and also depend on the structural details of the molecule-substrate(s) interfaces. To further simplify the problem, we will adopt the so called wide-band limit,[12] where the complex self-energies do not depend on the energy and are purely imaginary quantities. In this way, the corresponding matrices can be written as $\Sigma^{T/B} = (-\frac{i}{2})\gamma^{T/B}C^{T/B}$, where $C^{T/B}$ is a contact matrix defining which atoms of the molecule are in contact with the corresponding electrode and $\gamma^{T/B}$ is now a real number defining the energy scale associated with the molecule-contact coupling. With this, we bypass the need for calculating explicitly the electronic structure of the electrodes and the related self-energy matrices. Taking into account the experimental setup, we assume multiple contact points on the bottom (B) of the molecule (the region in closest contact with the Au substrate, see Figure 5d) and just one contact point on the top (T), the region in contact with the c-AFM tip during the transport measurements. We have checked that inclusion of a varying number of contact points only has a weak quantitative influence, inducing varying broadening of the transmission resonances, but without affecting the overall result, see Fig. S47.

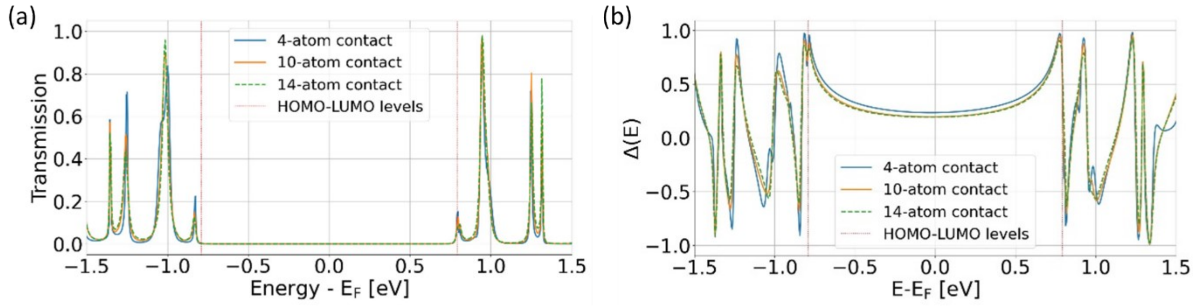

*Figure S47. (a) Influence of a varying number of contacted atoms on the bottom part of the helical NG **1** (the graphene flake in close contact with the Au(111) surface) on the quantum mechanical transmission function. Only a weak increase in the broadening of the transmission resonances is found, so that all calculations reported in the main text were carried out for a 14-atom contact case. (b) Same as in (a), but for the spin asymmetry factor (defined in the main text). Here, again, there are only small quantitative changes.*

.

7. References

[1] Y. S. Kim, S. J. Jeong, H. W. Lee, J. Kim, S. E. Lee, Y. K. Kim, S. S. Yoon. *Luminescence* **2016**, *31*, 1031-1036.

[2] X. Yang, X. Dou, K. Müllen, *Chem. Asian J.* **2008**, *3*, 759-766.

[3] Mueller, U.; Förster, R.; Hellmig, M.; Huschmann, F. U.; Kastner, A.; Malecki, P.; Pühringer, S.; Röwer, M.; Sparta, K.; Steffien, M.; et al. *Eur. Phys. J. Plus* **2015**, *130*, 141.

[4] Sparta, K. M.; Krug, M.; Heinemann, U.; Mueller, U.; Weiss, M. S. XDSAPP2.0. *J. Appl. Cryst.* **2016**, *49*, 1085-1092.

[5] Kabsch, W. XDS. *Acta Cryst. D* **2010**, *66*, 125-132.

[6] Sheldrick, G. *Acta Cryst. C* **2015**, *71*, 3-8.

[7] Gaussian 16, Revision C.01, M. J. Frisch, G. W. Trucks, H. B. Schlegel, G. E. Scuseria, M. A. Robb, J. R. Cheeseman, G. Scalmani, V. Barone, G. A. Petersson, H. Nakatsuji, X. Li, M. Caricato, A. V. Marenich, J. Bloino, B. G. Janesko, R. Gomperts, B. Mennucci, H. P. Hratchian, J. V. Ortiz, A. F. Izmaylov, J. L. Sonnenberg, D. Williams-Young, F. Ding, F. Lipparini, F. Egidi, J. Goings, B. Peng, A. Petrone, T. Henderson, D. Ranasinghe, V. G. Zakrzewski, J. Gao, N. Rega, G. Zheng, W. Liang, M. Hada, M. Ehara, K. Toyota, R. Fukuda, J. Hasegawa, M. Ishida, T. Nakajima, Y. Honda, O. Kitao, H. Nakai, T. Vreven, K. Throssell, J. A. Montgomery, Jr., J. E. Peralta, F. Ogliaro, M. J. Bearpark, J. J. Heyd, E. N. Brothers, K. N. Kudin, V. N. Staroverov, T. A. Keith, R. Kobayashi, J. Normand, K. Raghavachari, A. P. Rendell, J. C. Burant, S. S. Iyengar, J. Tomasi, M. Cossi, J. M. Millam, M. Klene, C. Adamo, R. Cammi, J. W. Ochterski, R. L. Martin, K. Morokuma, O. Farkas, J. B. Foresman, and D. J. Fox, Gaussian, Inc., Wallingford CT, **2016**.

[8] Grimme, S., Ehrlich, S. & Goerigk, L. *J. Comput. Chem.* **2011**, *32*, 1456–1465.

[9] DFTB+, a software package for efficient approximate density functional theory based atomistic simulations. *J. Chem. Phys.* **2020**, *152*, 124101.

[10] S. Grimme, J. Antony, S. Ehrlich, H. Krieg, *J. Chem. Phys.* **2010**, *132*, 154104.

[11] A. Tkatchenko, M. Scheffler, *Phys. Rev. Lett.* **2009**, *102*, 073005.

[12] C. J. O. Verzijl, J. S. Seldenthuis, J. M. Thijssen, *J. Chem. Phys.* **2013**, *138*, 094102.

[13] S. Varela, M. Peralta, V. Mujica, B. Berche, E. Medina. *SciPost Phys.* **2023**, *6*, 044.

[14] R. Gutierrez, E. Díaz, R. Naaman, G. Cuniberti, *Phys. Rev. B* **2012**, *85*, 081404.

[15] A.-M. Guo, Q.-f. Sun, *Phys. Rev. Lett.* **2012**, *108*, 218102.

[16] E. Díaz, F. Domínguez-Adame, R. Gutierrez, G. Cuniberti, V. Mujica, *J. Phys.Chem. Lett.* **2018**, *9*, 5753-5758.

[17] F. Evers, A. Aharony, N. Bar-Gill, O. Entin-Wohlman, P. Hedegård, O. Hod, P. Jelinek, G. Kamieniarz, M. Lemeshko, K. Michaeli, V. Mujica, R. Naaman, Y. Paltiel, S. Refaely-Abramson, O. Tal, J. Thijssen, M. Thoss, J. M. van Ruitenbeek, L.Venkataraman, D. H. Waldeck, B. Yan, L. Kronik, *Adv. Mater.* **2022**, *34,* 2106629.
